# How do non-independent host movements affect spatio-temporal disease dynamics? Partitioning the contributions of spatial overlap and correlated movements to transmission risk

**DOI:** 10.1186/s40462-025-00539-4

**Published:** 2025-02-26

**Authors:** Juan S. Vargas Soto, Justin R. Kosiewska, Dan Grove, Dailee Metts, Lisa I. Muller, Mark Q. Wilber

**Affiliations:** School of Natural Resources, University of Tennessee Institute of Agriculture, Knoxville, TN 37996 USA

**Keywords:** Transmission, Contact, Wildlife disease, Spatial overlap, Utilization distribution, Animal movement

## Abstract

**Background:**

Despite decades of epidemiological theory making relatively simple assumptions about host movements, it is increasingly clear that non-random movements drastically affect disease transmission. To better predict transmission risk, theory is needed that quantifies the contributions of both fine-scale host space use and non-independent, correlated host movements to epidemiological dynamics.

**Methods:**

We developed and applied new theory that quantifies relative contributions of fine-scale space use and non-independent host movements to spatio-temporal transmission risk. Our theory decomposes pairwise spatio-temporal transmission risk into two components: (i) spatial overlap of hosts—a classic metric of spatial transmission risk – and (ii) pairwise correlations in space use – a component of transmission risk that is almost universally ignored. Using analytical results, simulations, and empirical movement data, we ask: under what ecological and epidemiological conditions do non-independent movements substantially alter spatio-temporal transmission risk compared to spatial overlap?

**Results:**

Using theory and simulation, we found that for directly transmitted pathogens even weak pairwise correlations in space use among hosts can increase contact and transmission risk by orders of magnitude compared to independent host movements. In contrast, non-independent movements had reduced importance for transmission risk for indirectly transmitted pathogens. Furthermore, we found that if the scale of pathogen transmission is smaller than the scale where host social decisions occur, host movements can be highly correlated but this correlation matters little for transmission. We applied our theory to GPS movement data from white-tailed deer (*Odocoileus virginianus*). Our approach predicted highly seasonally varying contributions of the spatial and social drivers of transmission risk – with social interactions augmenting transmission risk between hosts by greater than a factor of 10 in some cases, despite similar degrees of spatial overlap. Moreover, social interactions could lead to a distinct shift in the predicted locations of transmission hotspots, compared to joint space use.

**Conclusions:**

Our theory provides clear expectations for when non-independent movements alter spatio-temporal transmission risk, showing that correlated movements can reshape epidemiological landscapes, creating transmission hotspots whose magnitude and location are not necessarily predictable from spatial overlap.

**Supplementary Information:**

The online version contains supplementary material available at 10.1186/s40462-025-00539-4.

## Background

Individual movement is a critical factor influencing wildlife disease dynamics [1, 2]. Movement determines encounters with other individuals of the same species, other species, or pathogens in the environment [3, 4]. These encounters are necessary for the transmission of infectious diseases, and efforts have sought to identify where they occur, how often, and how they are influenced by environmental and social drivers [5–7]. Formally linking social factors, environmental factors, animal movement, contact, and pathogen transmission would improve our ability to predict and prevent outbreaks and represent a significant advance for management of wildlife diseases. Nevertheless, understanding how these processes interact at an individual scale requires detailed movement information and theory to translate movement into an epidemiological context.

Most epidemiological theory is built upon the assumption of independent host movements, and there is little theory that quantifies how non-independent, correlated movements affect contact and transmission risk. Despite empirical work quantifying how correlated and social movements can affect contact and transmission landscapes [e.g., 8, 9, 10], we lack models that isolate the role of social interactions on spatio-temporal force of infection (FOI, the risk of transmission experienced by a host per unit time). This limits our ability to ask a key question for predicting spatio-temporal transmission risk on real-world landscapes: how do non-independent movements affect spatio-temporal infection risk, compared to spatial overlap? In other words, under what conditions are patterns of animal space use sufficient for predicting contact and transmission and when do correlations in animal movements driven by social dynamics alter spatio-temporal transmission patterns relative to space use alone? By space use, we specifically mean the probabilistic surface of space use for one individual, irrespective of other individuals on a landscape. By non-independent, correlated movements, we refer to any social process such as territoriality or gregariousness that could change the probability of two individuals being in the same place at the same or different times. If correlations in animal movement generally have negligible effects on transmission once space use is known, then epidemiological landscapes [*sensu* 2] can be largely predicted by understanding patterns of resource selection. However, recent studies have shown that spatial transmission risk can be highly localized [11] and is not necessarily predicted by animal space use [12]. We hypothesize that non-independent animal movements can (at least partially) account for these observations. We develop a modeling approach to rigorously test this hypothesis and systematically quantify the contribution of space use and non-independent movements to spatio-temporal transmission risk.

Recent developments at the interface of movement and disease ecology leverage high-resolution animal tracking data to gain insight into contact among individuals and disease transmission [13–15]. For example, movement-driven modeling of spatio-temporal infection risk (MoveSTIR) builds dynamic contact networks from movement data to estimate individual risk of infection across space and time [14]. MoveSTIR provides a theoretical foundation to translate contacts into the epidemiological currency of FOI. These studies have highlighted the importance of individual heterogeneity and temporal scale for epidemiology, particularly how indirect contact—individuals at the same place at different times—can significantly reshape contact and transmission networks [13, 15]. Current approaches are based on occurrence, rather than range, distributions [in the terminology of 16] – meaning they only consider where animals were and not where they *potentially* could be. This approach makes it difficult to systematically link encounters with environmental drivers, and to predict how social or environmental changes affect contact and transmission. Moreover, while MoveSTIR rigorously translates observed movement trajectories into metrics of epidemiological risk, it does not provide a way to partition the contributions of spatial overlap and non-independent host movements to epidemiological risk. Such a partition is needed to quantify and subsequently predict the ecological and epidemiological conditions where spatial and social drivers differentially affect transmission risk.

To address these limitations of MoveSTIR, it is useful to probabilistically consider spatio-temporal contact dynamics using utilization distributions (UDs). The UD represents the probability—transient or long-term [17, 18]—of an organism using some area [19]. The high spatial and temporal resolution of modern tracking data serves to build UDs based on biologically realistic movement models [20, 21], and to link them with underlying resources [22]. Additionally, combining individual UDs informs about pairwise interactions, by quantifying home range overlap [23], or estimating the expected location and rate of encounters [24], which could serve to infer transmission risk [24–26]. Moreover, because UDs can be directly linked to environmental drivers of movement [27], they could be used for prospective analyses, to predict contact and transmission in novel environments, or to understand cascading effects of environmental and social perturbations from individual movement to population and landscape-level disease transmission. Thus, UDs provide a general and intuitive method for i) linking contacts closely with environmental context (i.e., environment $\rightarrow$ UD $\rightarrow$ contact) and ii) predicting potential contacts beyond observed movement trajectories. UDs also help clarify a distinction we use between processes affecting animal movements and the resulting probabilistic space use. Animal movements on a landscape are driven by myriad spatial (e.g., the selection of resources) and social (e.g., tending a potential mate) processes that together generate a probabilistic surface of space use – the UD.

However, current contact metrics based on UDs have two limitations. First, they typically focus only on direct interactions, ignoring temporal dynamics related to indirect interactions that are especially relevant for epidemiological processes [15]. Second, they consider independently moving animals, effectively assuming that any correlation in animal movement is unimportant for contact once space use is known. The Conditional Distribution of Encounters (CDE) [24], for example, estimates local probabilities of encounter as a product of individual UDs, assuming that individuals move independently. While a useful simplification, social interactions like territoriality or gregariousness can invalidate this assumption [28, 29]. In these cases, temporal correlations in space use could increase or decrease the probability of encounter expected given independent movement [8, 10]. Moreover, direct interactions do not necessarily equate to *epidemiological contacts*, which comprise contact formation and duration, as well as pathogen shedding, decay, and acquisition [30]. As some pathogens can persist in the environment for months or years (e.g. anthrax, chronic wasting disease–CWD), ignoring these processes could severely underestimate transmission risk [13–15]. An analytical framework is needed that combines utilization distributions, non-independent movements, and direct and indirect epidemiological interactions to quantitatively assess how these factors jointly shape spatio-temporal FOI and disease dynamics on real landscapes.

Here, we develop a model we refer to as Probabilistic MoveSTIR (PMoveSTIR) to address a major knowledge gap at the interface of movement and disease ecology: under what ecological and epidemiological conditions do non-independent host movements affect spatio-temporal transmission dynamics? We derive a general model that decomposes the contributions of transient, spatially heterogeneous UDs and a novel metric we refer to as the “pairwise correlation surface” that quantifies the contribution of non-independent host movements to spatio-temporal transmission risk. We use analytical results to examine how the scale of pathogen transmission, the scale of host social decisions, pathogen persistence in the environment, and host movement patterns affect transmission risk. We apply our model to empirical movement data for white-tailed deer (*Odocoileus virginianus*) to examine how realistic, non-independent movements can alter the magnitude and configuration of transmission hotpots on epidemiological landscapes.

## Methods

### Model development—review of MoveSTIR

PMoveSTIR builds on the MoveSTIR model [14] and formally links UDs, direct and indirect contacts, non-independent movements, and spatial estimates of FOI. Essentially, we want to know, for two individuals *i* and *j* moving and interacting across a landscape, what is the expected FOI *i* experiences from *j*, across space and time?

As in MoveSTIR, we assume that transmission happens by an infected host depositing pathogen into the environment and another host picking that pathogen up. Deposition and acquisition can represent a range of processes, from coughing and inhaling in a matter of seconds, to depositing parasite eggs or larvae in the environment and another individual consuming these days or weeks later. Considering transmission through deposition and acquisition clearly links direct and indirect transmission along a continuum [14], and it encompasses standard density-dependent transmission as a special case [31].

As derived in [14], MoveSTIR defines the pairwise FOI host *j* exerts on host *i* in location *x* at time *t* as [14] (see Appendix 1 for a full derivation of equation 1)

1 $$\begin{aligned} h_{i \leftarrow j}(t, x)= \int _{-\infty }^{t} &\underbrace{\beta '}_{\text {Acquisition}} \underbrace{\delta _{x_j(u)}(x)}_{\begin{array}{c} \text {Contact:} \\ j \text { in location } x \text { at } u \end{array}} \nonumber \\ &\underbrace{\lambda \delta _{I_j(u)}(I)}_{\text {Deposition}} \underbrace{\Theta (t - u)}_{\text {Pathogen survival}} du \end{aligned}$$

The term $x_j(u)$ is the location of individual *j* at time *u* and $\delta _{x_j(u)}(x)$ is an indicator function that is one if host *j* is in location *x* at time *u* and zero otherwise. A location *x* has some area $A_x$. The parameter $\beta '$ is the rate at which host *i* acquires pathogen within location *x* and can be re-written as ${\tilde{\beta }} / A_x$, where ${\tilde{\beta }}$ can be considered a “search efficiency” term, with units area/time (e.g., $m^2 / day$), and $A_x$ gives the area of location *x* where transmission can occur (henceforth the area of transmission). In equation 1, we assume that the likelihood of contact is uniform within location *x* [14, 32]. The area $A_x$ of location *x* is a biological property of the pathogen separate from the movement ecology of the host and effectively defines how close individuals need to be before transmission can occur. While MoveSTIR considers different types of contacts based on the distance between individuals in continuous space [14], here we assume that transmission occurs within grids on a landscape where the area of a grid cell $A_x$ is determined by the epidemiology of the pathogen of interest – contact occurs when two individuals are in the same grid cell (but see Appendix S2). How individuals move is independent of $A_x$, but how pathogens are transmitted depends on $A_x$. While this grid approach is less general than a distance-based approach, it is consistent with how many individual-based models capture direct or indirect transmission on landscapes [e.g. 33, 34, 35] and also leads to many useful conceptual results [e.g., 4].

The parameter $\lambda$ is the per capita pathogen deposition rate. We assume that $\lambda$ is constant and does not depend on time since infection. The variable $\delta _{I_j(u)}(I)$ is an indicator function that is one if host *j* is infected at time *u* and zero otherwise. The function $\Theta (t - u)$ is a pathogen survival function that gives the probability that pathogens deposited at time $u < t$ are still transmissible at time *t*.

It is also useful to consider a consequence of some of our assumptions underlying equation 1. The parameter $\lambda$ is a per capita shedding rate and is not scaled by area. Over a unit of time, infected hosts will shed a finite amount of pathogen into the location *x* that they are occupying. Equation 1 assumes that this pathogen instantaneously and uniformly fills the location *x* such that there is a density of pathogen $\lambda dt \Theta (dt) / A_x$ in *x* after *dt* time units. As $A_x$ approaches zero, the deposited pathogen becomes infinitely dense and, because we assume there is no pathogen depletion upon acquisition, FOI goes to infinity. While this limit is logically consistent with density-dependent transmission and no pathogen depletion, it is biologically nonsensical because a discrete host cannot occupy, shed, and acquire pathogen in an infinitesimally small area. Thus, there is a biological lower bound on $A_x$ that ensures that at least one host can fit into *x*. In what follows we will always consider that $A_x$ at minimum can encompass the depositing host and is often larger than the host (e.g., consider the 2 m radius for transmission of the SARS-CoV-2 virus during the COVID-19 pandemic).

### Model development—linking utilization distributions to transmission through PMoveSTIR

Our core PMoveSTIR equation emerges from equation 1. We give the derivation in Appendix 3, and here we present the key result from which we can gain biological insight. Specifically, the expected FOI $h^*_{i \leftarrow j}(x):= E[h_{i \leftarrow j}(x)]$ given probabilistic space use and an assumption of statistical stationarity in host movement is

2 $$\begin{aligned} \begin{aligned} h^*_{i \leftarrow j}(x)&= \beta ' \lambda [ \underbrace{p_i(x)p_j(x) \int _0^{\infty } \Theta (s) ds}_{\begin{array}{c} \text {FOI contribution from} \\ \text {spatial overlap} \end{array}} \\&+ \underbrace{\sigma _i(x) \sigma _j(x) \int _{0}^{\infty } Cor(\delta '_{x_i(k)}(x), \delta '_{x_j(k - s)}(x)) \Theta (s) ds}_{\begin{array}{c} \text {FOI contribution from} \\ \text {non-independent movement} \end{array}}]. \end{aligned} \end{aligned}$$

“Stationarity” says that host’s mean space use is not changing over time and their covariance in space use only depends on time lags, not absolute times. In equation 2, the quantity $p_i(x)$ is the stationary probability of host *i* using location *x* and $\sigma _i(x) = \sqrt{p_i(x)(1 - p_i(x))}$ is the standard deviation in probability of host *i* using location *x* (defined similarly for host *j*). The term $Cor(\delta '_{x_i(k)}(x), \delta '_{x_j(k - s)}(x))$ is the (lagged) correlation between the occupancy random variables $\delta '_{x_i(k)}(x)$ and $\delta '_{x_j(k - s)}(x)$ for any time *k* where the time lag is *s*. Given the assumption of statistical stationarity, the correlation between the occupancy random variables of host *i* and host *j* does not depend on absolute time *k* or $k - s$, but just the time lag *s*. This is the quantity we refer to as the “pairwise correlation surface”. Henceforth, we let $\rho (x,s) = Cor(\delta '_{x_i(k)}(x), \delta '_{x_j(k - s)}(x))$.

The pairwise correlation surface in equation 2 is strongly related to the biology of host movements and the epidemiology of pathogen transmission. A higher correlation term means that given host *i* is in location *x* there is a higher probability that host *j* is also in location *x*. For a correlation term equal to one, hosts *i* and *j* are both in location *x*. Social interactions between hosts and the area of transmission $A_x$ will influence $\rho (x,s)$.

Using equation 2, we can now ask: how much does the term $\sigma _i(x) \sigma _j(x) \int _{0}^{\infty } \rho (x, s) \Theta (s) ds$ contribute to FOI for different areas of transmission, social dynamics, and epidemiological scenarios? Therefore, we will focus on the ratio between non-independent movement contributions to FOI and spatial overlap (i.e., the correlation-spatial overlap ratio, *CSR*(*x*)): $CSR(x) = \frac{| \sigma _i(x) \sigma _j(x) \int _{0}^{\infty } \rho (x, s) \Theta (s) ds |}{p_i(x) p_j(x) \int _{0}^{\infty } \Theta (s) ds}$. The vertical bars indicate absolute values.

### Analytical analysis: the effect of non-independent movements on FOI for uniform space use and direct contact

Assume hosts are moving across a landscape of area $A_{tot}$. If they are using space uniformly, the probability of using any location *x* with area $A_x$ is constant across space and among individuals. If pathogen decay is rapid relative to host movement (direct transmission), we can update equation 2 as (derivation in Appendix 4)

3 $$\begin{aligned} \begin{aligned} h^*_{i \leftarrow j}(x) = \beta ' \lambda T \left[ \underbrace{\frac{A_x}{A_{tot}}\frac{A_x}{A_{tot}}}_{\begin{array}{c} \text {Contribution due} \\ \text {to spatial overlap} \end{array}} + \underbrace{\frac{A_x}{A_{tot}}(1 - \frac{A_x}{A_{tot}}) \rho (x, s=0)}_{\begin{array}{c} \text {Contribution due} \\ \text {to non-independent movement} \end{array}} \right] . \end{aligned} \end{aligned}$$

where *T* is the (short) duration of time that a pathogen is viable following deposition and $\rho (x, s = 0)$ is the correlation in two hosts’ space use with a time lag of 0.

The *CSR*(*x*) is simply

4 $$\begin{aligned} CSR(x) = (\frac{A_{tot}}{A_{x}} - 1) | \rho (x, s=0) | \end{aligned}$$

While $\rho (x, s=0)$ is not fixed for $A_x$, we can explore how varying values of $A_x / A_{tot}$ and $\rho (x, s=0)$ affect *CSR*(*x*) because i) $\rho (x, s=0)$ is driven by host parameters (e.g., mechanisms of social attraction) that are independent of $A_x$ (a pathogen parameter) and ii) $A_x / A_{tot}$ can change through $A_{tot}$ while $A_{x}$ remains constant.

Interpreting equation 4 for $\rho (x, s=0) > 0$ (i.e., non-independent movements related to social attraction), we see that when the area of transmission $A_x$ is small *relative* to the total area that a host can occupy $A_{tot}$, even small levels of correlation $\rho (x, s=0)$ can result in non-independent movements having orders of magnitude larger contribution to FOI for direct transmission than spatial overlap. This finding makes sense but is almost never considered in epidemiological models. If the area of transmission $A_x$ is small relative to $A_{tot}$ and hosts are moving randomly, there is a very low chance that hosts will be in an area of transmission together at the same time. Non-independent movements related to social attraction significantly increase this chance, even when correlation is low. In contrast, if a host’s home range is similar in size to the area of transmission, then the importance of non-independent movement relative to spatial overlap becomes minimal. Given that transmission for many directly transmitted pathogens require hosts to be in relatively close contact, we would expect many empirical systems to be in the region where $A_x<< A_{tot}$, meaning that even small levels of $\rho (x, s=0) > 0$ can have significant effects on FOI compared to spatial overlap alone.

Equation 3 also provides intuitive insight into the role of social avoidance ($\rho (x, s=0) < 0$) on FOI. Because FOI must be positive, there is an inherent lower bound on $\rho (x, s=0)$. From equation 3, the following inequality must hold: $\rho (x, s=0) > \text {max}\left( - \frac{A_x}{A_{tot}} / (1 - \frac{A_x}{A_{tot}}), -1\right)$ (where −1 is included strictly because correlation is bounded between −1 and 1). Plugging $|-\frac{A_x}{A_{tot}} / (1 - \frac{A_x}{A_{tot}})|$ into equation 4, the maximum *CSR*(*x*) given social avoidance is 1. In other words, for any ratio $A_x / A_{tot}$ and direct transmission, non-independent movement related to social avoidance can at most contribute as much as spatial overlap to FOI. Because of this result, we focus on non-independent movement related to social attraction ($\rho (x, s=0) > 0$) for the remainder of this study (henceforth just “non-independent movement”).

### Using models to test the contribution of non-independent movements to FOI

We considered two different models to gain additional insight into how non-independent movement influences local FOI. We use these models to explore the relative contribution of non-independent host movements for different scales of pathogen transmission $A_x$, scales of host social decisions, strengths of host social interactions, and rates of pathogen decay. We use these models to examine our ability to empirically estimate the contribution of non-independent movements to FOI given limited movement data.

#### Model analysis, example 1: non-independent movements and the scale of social decisions

Here, we implement what we call a messy follow-the-leader model. This is a strategic model that is geared to honing our intuition regarding the relative contributions of space use and non-independent movements to FOI. The steps in the model are as follows. There are some number of individuals on a landscape that have a strict social hierarchy – individual 1 is dominant to individual 2, individual 2 is dominant to individual 3, and so on. Individuals can move between $N_h$ equal sized patches on the landscape, each with an area of 1 unit (units are arbitrary). At each time step, dominant individuals move first, randomly selecting the next patch to move to. Any subordinate individuals that were in the same patch will either follow the most dominant individual with probability $p_f$, or select a random patch with probability $1 - p_f$.

The area of transmission is assumed to be smaller than or equal to the 1 unit area of the patches where social dynamics and movement decisions occur. Specifically, the area of transmission for a pathogen is $1 / n_p$ of the area of a habitat patch. For simplicity, one can envision $n_p$ grid cells comprise each habitat patch and transmission can happen when two hosts in the same patch are also in the same grid cell. Once in a patch, we assume that individuals are randomly located within the patch. Under these conditions, non-independence in movement may result in epidemiologically relevant correlation in space use, but this will depend on the relative size of the grid cells (where transmission happens) and the patches (where social decisions are made).

**The scale of social decisions relative to the area of transmission** $A_x$

The messy follow-the-leader model has an analytical solution for correlation in movement for any two individuals at a time lag of 0 (derived in Appendix 5). The *CSR*(*x*) is

5 $$\begin{aligned} CSR(x) = \frac{p_f(N_h - 1)}{N_h(n_p - p_f) + p_f}. \end{aligned}$$

**Fig. 1:**
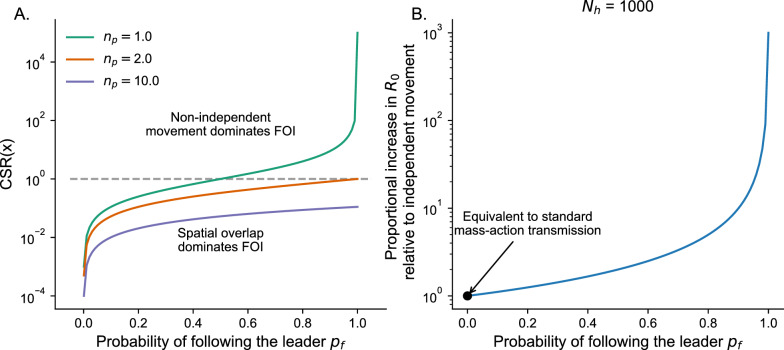
**A** The relative contribution of non-independent movements compared to spatial overlap on FOI (*CSR*(*x*)), as calculated by equation 5, for varying probabilities of following the leader $p_f$. The different color lines show different values of $n_p$, where the area of transmission relative to the area in which social decisions to follow the leader are made is $1 / n_p$. The dashed line shows a relative contribution of 1, meaning that non-independent host movements and spatial overlap contribute equally to FOI. **B** The effect of non-independent host movements as given by $p_f$ on $R_0$ in an SIR model (equation 6). The y-axis shows the increase in $R_0$ relative to an equivalent SIR model where $p_f = 0$ and hosts are completely uncorrelated in their movements and space use and are moving randomly in space (i.e., mass-action transmission). $N_h$ is the number of equally sized patches on the landscape that hosts move between

Equation 5 highlights two keys results. First, if the area of transmission $A_x = 1 / n_p \le 1 / 2$ (i.e., the area of transmission is half the size or less compared to the area over which hosts are making social decisions), then the contribution of non-independent movement to FOI can at most be equal to the contribution from spatial overlap. This happens when $N_h$ is large and $p_f = 1$ (equation 5, Fig. 1A). As $1 / n_p$ goes to zero, the *CSR*(*x*) goes to zero regardless of the probability of following the leader $p_f$ (Fig. 1A). If the social decisions are happening at a scale substantially larger than the pathogen’s area of transmission, then from the pathogen’s perspective hosts are moving randomly and correlation in movement does not matter for FOI.

Second, in stark contrast to the above result, if social decisions are happening at a similar scale as the area of transmission ($n_p = 1$), then there is always some $p_f$ for which non-independent space use will have a higher contribution to FOI compared to spatial overlap (Fig. 1A). For example, as $N_h$ gets very large and $n_p = 1$, meaning that the total area that hosts move on the landscape is large relative to the area of transmission, the *CSR*(*x*) approaches $p_f / (1 - p_f)$. If $p_f$ is greater that 0.5, then non-independent movement will matter more than spatial overlap for FOI, otherwise non-independent movement will matter less than spatial overlap (Fig. 1A).

**Non-independent movement and population-level disease dynamics**

For a pathogen invading the messy follow-the-leader model with Susceptible-Infected-Recovered dynamics (model described in Appendix 6), direct transmission, and $n_p = 1$, where $\beta$ is the transmission rate per patch, $\gamma$ is the per capita recovery rate, and *H* is the total number of susceptible individuals in the population, the fundamental recruitment number $R_0$ (i.e., the expected number of new infections produced over the lifetime of an average infected individual in a wholly susceptible population) is

6 $$\begin{aligned} R_{0}= & \dfrac{\beta (H - 1) [\frac{1}{N_h} + (1 - \frac{1}{N_h})\frac{p_f}{N_h(1 - p_f) + p_f}]}{\gamma }\nonumber \\= & \dfrac{\beta (H - 1) (\frac{1}{N_h(1 - p_f) + p_f})}{\gamma } \end{aligned}$$

Increasing $N_h$ for a fixed $p_f$ and $\beta$ ultimately reduces $R_0$ as hosts are moving over a larger landscape relative to the area of transmission, decreasing transmission risk. Increasing $p_f$ increases $R_0$ as individual movements are more correlated (Fig. 1B). When $p_f = 1$, $R_0 = (\beta ) (H - 1) / \gamma$ indicating that perfect correlation essentially reduces the landscape to a single patch, increasing transmission risk (Fig. 1B). In contrast, when $p_f = 0$ then $R_0 = \frac{\beta }{N_h} (H - 1) / \gamma$, illustrating that because hosts are moving randomly across the landscape $R_0$ is equivalent to mass action transmission on a landscape with area $N_h$ (Fig. 1B). Overall, equation 6 shows that non-independent movements directly affect epidemiological dynamics at the population-level by modifying $R_0$.

#### Model analysis, example 2: continuous-time movements and the effects of non-independent movement on direct and indirect transmission

In our second model, we quantify the relative contribution of non-independent movement to FOI compared to spatial overlap when hosts are moving in continuous space and time. We also move beyond direct transmission and examine how the *CSR*(*x*) is modulated by pathogen persistence in the environment.

We model two hosts that have the same home range center, but have different levels of social attraction to each other as they move within their home ranges. The hosts move following an Ornstein-Uhlenbeck (OU) process; i.e., they move randomly but are attracted back towards the center of their home range as they drift away [36, 37]. Specifically, we model the OU process as [36]

7 $$\begin{aligned} {\textbf{x}}(t + \Delta t) \sim MVN(\varvec{\mu } + e^{-{\textbf{B}}\Delta t}({\textbf{x}}(t) - \varvec{\mu }), \varvec{\Lambda } - e^{-{\textbf{B}}\Delta t} \varvec{\Lambda } e^{-\mathbf {B'}\Delta t} ) \end{aligned}$$

where MVN represents the probability density function of a multivariate normal distribution. The parameter ${\textbf{x}}(t)$ is the location vector at time *t*, $\varvec{\mu }(t)$ is the home range center vector (i.e., the point of attraction), ${\textbf{B}}$ is the drift matrix, and $\mathbf {\Lambda }$ is the variance-covariance matrix. We model a $4 \times 1$ vector ${\textbf{x}}(t)$ where the first two entries in the vector are the x and y locations of host 1 and the second two entries are the x and y locations of host 2. We model the drift matrix ${\textbf{B}} = c {\textbf{I}}$ where ${\textbf{I}}$ is the $4 \times 4$ identity matrix and *c* is a constant with units per time that specifies the tendency of an individual to return to the home range center $\varvec{\mu }$ following a departure. We model the variance-covariance matrix $\varvec{\Lambda }$ as

8 $$\begin{aligned} \varvec{\Lambda } = \begin{bmatrix} \sigma & 0 & 0 & 0 \\ 0 & \sigma & 0 & 0 \\ 0 & 0 & \sigma & 0 \\ 0 & 0 & 0 & \sigma \\ \end{bmatrix} \times \begin{bmatrix} 1 & 0 & \xi & 0 \\ 0 & 1 & 0 & \xi \\ \xi & 0 & 1 & 0 \\ 0 & \xi & 0 & 1 \\ \end{bmatrix} \times \begin{bmatrix} \sigma & 0 & 0 & 0 \\ 0 & \sigma & 0 & 0 \\ 0 & 0 & \sigma & 0 \\ 0 & 0 & 0 & \sigma \\ \end{bmatrix} \end{aligned}$$

where $\sigma$ is the standard deviation (units of distance) in space use for each host across each dimension and $\xi$ defines our social attraction coefficient ranging from 0 to 1 (unitless), which induces positive correlation in movement along the x and y axis for host 1 and host 2. The long-run stationary distribution of the OU process converges on $MVN(\varvec{\mu }, \varvec{\Lambda })$ [36], which is a well-defined distribution from which we can compute the components of equation 2 – marginal UDs for host 1 and host 2, standard deviations in space use, and the pairwise correlation surface (see Appendix 7 for an example).

**Fig. 2:**
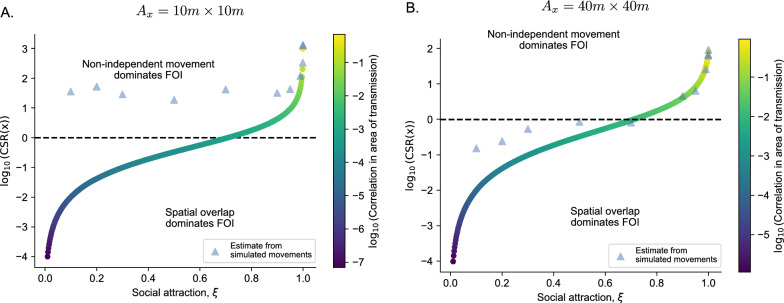
The relative contribution of non-independent host movement to FOI from direct transmission compared to spatial overlap for varying levels of social attraction $\xi$ for hosts moving following an OU process. The contributions of non-independent movement were calculated on (**A**) 10 m $\times$ 10 m grid cell and (**B**) 40 m $\times$ 40 m grid cells centered on the home range center. The dashed line indicates an equal contribution of non-independent movement and spatial overlap for FOI. The colors of the points indicate the correlation $\rho (x, s=0)$ in space use between the two hosts for a given value of social attraction $\xi$. Finally, the blue triangles are a result of statistically estimating the relative contribution of non-independent movement from 1 year of movement data from two hosts with a drift coefficient of $c = 0.05 \text { hour}^{-1}$ and $\sigma = 150\,m$

We examine how *CSR*(*x*) varies across different levels of social attraction $\xi$ within a 10 m by 10 m grid cell at the center of a host’s home range (specifically, this area of transmission might be relevant for pathogen transmission in white-tailed deer, but our general results are not sensitive to this specific area). We assume two hosts are moving according to an OU process with $\sigma = 150\,m$ and home range centers $\varvec{\mu } = [0 \hspace{0.1cm} 0 \hspace{0.1cm} 0 \hspace{0.1cm} 0]'$ (ignoring *c* for the moment as this rate does not matter for the long-term stationary probability). We see two main results that are consistent with results in previous sections. First, social attraction may be present (e.g., $\xi > 0$) and induce synchrony in host movements, but synchrony must be at the scale of transmission to matter for FOI (at least relative to spatial overlap, Fig. 2A). Spatial overlap dominates FOI even when social attraction is present ($0< \xi < 0.7$; Fig. 2). Second, non-independent movement can increase FOI relative to spatial overlap by orders of magnitude even when correlation within areas of transmission is quite low (Fig. 2A). For example, even when $\rho (x, s=0) = 0.005$ with a social attraction of $\xi = 0.93$, the contribution of non-independent movement to FOI was seven times larger than spatial overlap.

Since low levels of spatial correlation in areas of transmission can lead to large increases in FOI, small correlations matter. However, small correlations are also hard to estimate empirically from finite movement data and small areas of transmission. To demonstrate this, we used our OU model with a drift coefficient such that host step lengths were roughly consistent with white-tailed deer in western Tennessee, USA ($c = 0.05 \text { hour}^{-1}$ yielding an average 10 min step-length in movement of approximately 50 m). We then asked how consistently we can recapture $\rho (x, s=0)$ and *CSR*(*x*) with just one year of empirical data and $A_x = 10\text {m} \times 10\text {m}$ (where the true $\rho (x, s=0)$ and *CSR*(*x*) is known from the exact analysis of the multivariate normal OU process). We estimated $\rho (x, s=0)$ by calculating the correlation coefficient of the 0/1 occupancy vectors of the two simulated host trajectories – where “occupancy” means that a host is in location *x* centered on the home range center. With (simulated) fine-scale movement data, we see that while we can re-capture the true *CSR*(*x*) when $\rho (x, s=0) > 0.05$, we consistently overestimate the contribution of non-independent movement to FOI when $\rho (x, s=0) < 0.05$ (Fig. 2A). The bias arises because the amount of movement data we have affects how precisely we can estimate $\rho (x, s=0)$, which can drastically affect our inference on *CSR*(*x*). However, increasing the potential area of transmission to 40 m $\times$ 40 m can allow us to estimate $\rho (x, s=0)$ with less bias from more realistically collected durations of movement data (Fig. 2B). Ultimately, randomization approaches [e.g., 38] that allow you to build null distributions based on non-independent movements will likely be important when assessing the relative contributions of non-independent movements to FOI from finite, empirical movement data (we use these approaches in examples below).

**Contribution of indirect transmission**

We have thus far focused on the contribution of non-independent movements to FOI given direct transmission. However, many pathogens can persist for a non-trivial amount of time in the environment and indirect transmission can play an important role for FOI [13, 15]. Using our OU model defined above, we ask: how does indirect transmission affect the relative contribution of non-independent movement to FOI compared to spatial overlap?

We return to equation 2 and let the pathogen survival function $\Theta (s)$ be a step function where survival of the pathogen is 1 up to time *T* and 0 thereafter. When *T* is small (relative to host residence times), we recover direct transmission as discussed above. When *T* is large, indirect contacts (i.e., hosts are in the same place but at different times) contribute more to transmission.

Consider the OU process with $\sigma = 150\,m$ and $\varvec{\mu } = [0, 0, 0, 0]'$. We vary the drift coefficient *c* from $0.005 \text { hour}^{-1}$ to $1 \text { hour}^{-1}$ and the social attraction coefficient from $\xi = 0.5, 0.9, 0.9999$. As we show in Appendix 8, given a stationary OU movement process, we can exactly calculate $\rho (x, s)$ for any lag *s* at any location *x* with area $A_x$. For simplicity, we again focus on a 10 m $\times$ 10 m area centered on ($x=0$, $y=0$) and test on how pathogen survival time *T* (i.e., accounting for indirect transmission) effects *CSR*(*x*).

**Fig. 3:**
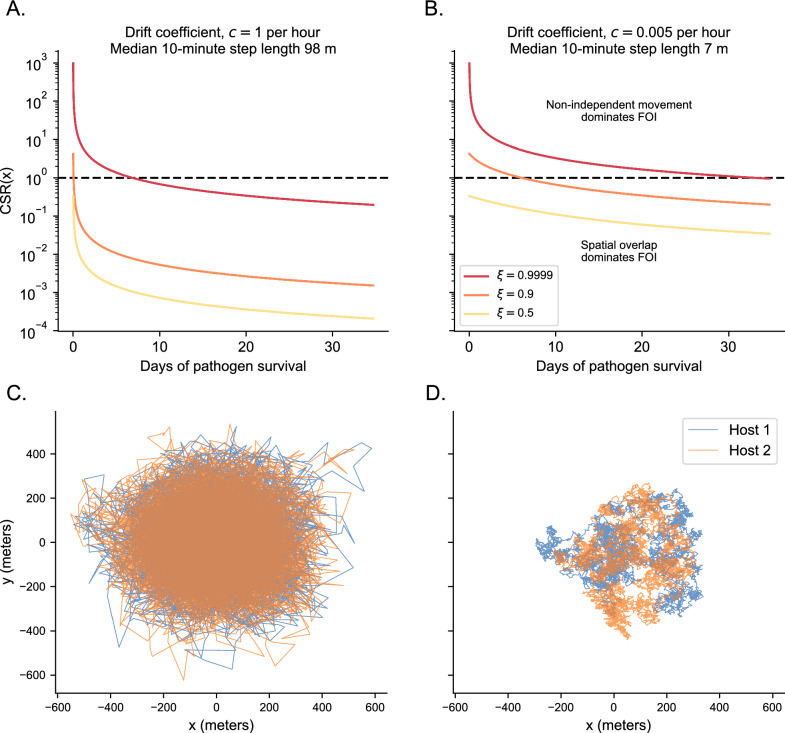
**A** The correlation-spatial overlap ratio (*CSR*(*x*)) for two hosts moving according to an OU process with $\sigma = 150 m$ with an attracting point at (0, 0). The different colored lines show different levels of social attraction between the two hosts, $\xi$. The dashed line shows when non-independent movement and spatial overlap have the same contribution to FOI. The drift coefficient is $c = 1 \text { hour}^{-1}$. **B** Same as A., but with a drift coefficient of $c = 0.005 \text { hour}^{-1}$. **C** Example trajectories from two hosts moving with $c = 1 \text { hour}^{-1}$ and $\xi = 0.9$. Locations are recorded every 10 min and trajectories are over 60 days. **D.** Same as C., but with $c = 0.005 \text { hour}^{-1}$

The key result is that the importance of non-independent movement to FOI decreases as the pathogens persist for longer in the environment, with transmission eventually being dominated by spatial overlap if pathogens persist for long enough (Fig. 3A). While this relationship generally holds, its strength is affected by the degree of autocorrelation that individual hosts have in their own movement trajectories (Fig. 3B). The reason is because social attraction (mediated by $\xi$ and ultimately $\rho (x, s)$) puts two hosts in the same area of transmission at the same time and autocorrelation in movement (mediated by *c*) keeps them in this area for longer. Thus, the augmentation of FOI due to non-independent movement when considering direct transmission (i.e, the left-hand side of the curves in Fig. 3B) can persist for varying degrees of indirect transmission as well. However, our theory generally shows that the transmission of longer-lived pathogens depends substantially less on non-independent movements than short-lived pathogens.

### Empirical application—white-tailed deer

Informed by the theory developed in PMoveSTIR, we tested the role of spatial overlap and non-independent movements on potential transmission risk in a real system, using GPS-tracking data for white-tailed deer captured at Ames AgResearch Center and Lone Oaks Farms, two properties separated by 25 km in west Tennessee, USA. In 2023 and 2024, 66 deer were captured and equipped with GPS collars that recorded fixes every 30 min (Lotek LiteTrack Iridium 420 and and 420+, Newmarket, Ontario, Canada; IACUC # 2850-1021; Tennessee Wildlife Resources Agency capture permit: 3059). There were 254 unique pairs of individuals that had spatial-temporal overlap (i.e., had overlapping collar times and overlapping 100% minimum convex polygons), allowing for an analysis of the contribution of joint space use and non-independent movements to transmission risk. Based on deer biology at our site, we separated a year into four distinct seasons: gestation (February - May), fawning (June - July), lactation (August - October), and rut (November - January), yielding 388 pair by season combinations (we truncated movement data in August 2024 for this analysis).

We asked three questions. First, how much do white-tailed deer social interactions augment FOI compared to spatial overlap, given an area of transmission $A_x = 40\text {m} \times 40 \text {m}$ and a directly transmitted pathogen (e.g., a pathogen like SARS-CoV-2)? For each pair by season combination, we temporally aligned trajectories and interpolated trajectories to 10 min fixes [following 15]. Over the space that individuals were jointly moving, we gridded the landscape into 40 m $\times$ 40 m grid cells and assumed that transmission of a SARS-CoV-2-like pathogen could only occur when hosts were sharing a cell. We chose this scale based on theoretical analysis in the previous sections—while transmission from a pathogen like SARS-CoV-2 likely occurs at a smaller scale (e.g., 10 m $\times$ 10 m or smaller), we are empirically limited to the scale which we can reliably estimate correlation surfaces. We chose 40 m $\times$ 40 m as this was the smallest scale that we could estimate a relatively complete correlation surface over the landscape deer were moving, without interpolation. For each grid, we computed the occupancy vectors for both individuals (1 the individual was in the cell, 0 the individual was not in the cell at time *t*) and computed the correlation coefficients between these occupancy vectors. This provided an estimate of $\rho (x, s=0)$ where *x* is a grid cell. If individuals never visited a cell, no correlation was calculated. To help minimize potential bias based on the arbitrary boundaries of cells, we computed five different placements of the boundaries for each cell and computed the average the resulting correlation estimates. We then computed UDs for each host using kernel density estimates (KDE) for each host in the season on the same gridded landscape, providing estimates of $p_i(x)$ and $p_j(x)$. We thinned trajectories every five hours to mitigate the effects of autocorrelation. These KDE-based UDs were similar to those calculated by an autocorrelated KDE method [39] and were far faster to compute. For each pair by season combination, we then computed the average per grid cell *CSR*(*x*) across all cells where we could estimate $\rho (x, s=0)$. Ultimately, this yielded 388 average *CSR*(*x*) estimates.

Second, we asked: Are known changes in deer social interactions reflected in seasonal changes in *CSR*(*x*) values? We would expect social interactions between deer to change over biological seasons, and thus the contribution of non-independent movements to FOI to also change. For example, social interactions between females in the same matrilineal group are predicted to be strong during gestation, but diminish during fawning and lactation [10, 40]. We focused on two pairs of individuals that showed strong evidence of social interactions: a female–female pair and a male-male pair. We used the same approach as above to compute per cell *CSR*(*x*) values, with the key difference being that we also used a randomization approach to test whether the *CSR*(*x*) were significantly different than we would expect from independent movement. Following [38], we randomized the temporal stamps of each individual by season movement trajectory by days (i.e., keeping timestamps the same within days, but randomly shuffling the day labels) and then recomputed *CSR*(*x*). This effectively removed most temporal dependencies between trajectories allowing us to ask about expected *CSR*(*x*) values with independent movement. We performed 200 randomizations per pair per season to compute our null distributions.

Finally, we examined whether non-independent movements changed the locations of hotspots on epidemiological landscapes compared to joint space use. Where FOI is high due to shared space use, correlation in space use might yield similar hotpots. In contrast, if locations of strong correlation in space use are unrelated to areas with a high probability of space use, this could lead to a transmission landscape where transmission hotspots are not predicable from space use alone. To test this, we again focused on the female–female and male-male pairs mentioned above, but only during gestation. For each grid cell *x* where we were able to estimate $\rho (x, s=0)$, we computed the spatial contribution to FOI $\propto p_i(x) p_j(x)$ and ranked each cell by its magnitude. We then computed the total contribution of space use and non-independent movement to FOI ($\propto p_i(x) p_j(x) + \sigma _i(x) \sigma _j(x) \rho (x, s=0)$) for each grid cell *x* and ranked the magnitudes. We calculated the Kruskal–Wallis test statistic *KW* that compares rank distributions. To generate a null distribution of *KW* statistics, we randomized each host’s trajectory 1000 times using the same approach described above and compute the same *KW* statistic each time. We expected our observed test statistic *KW* to be significantly greater than the randomized null distribution if non-independent movement was reshaping the transmission landscape compared to spatial overlap alone.

## Results

**Fig. 4:**
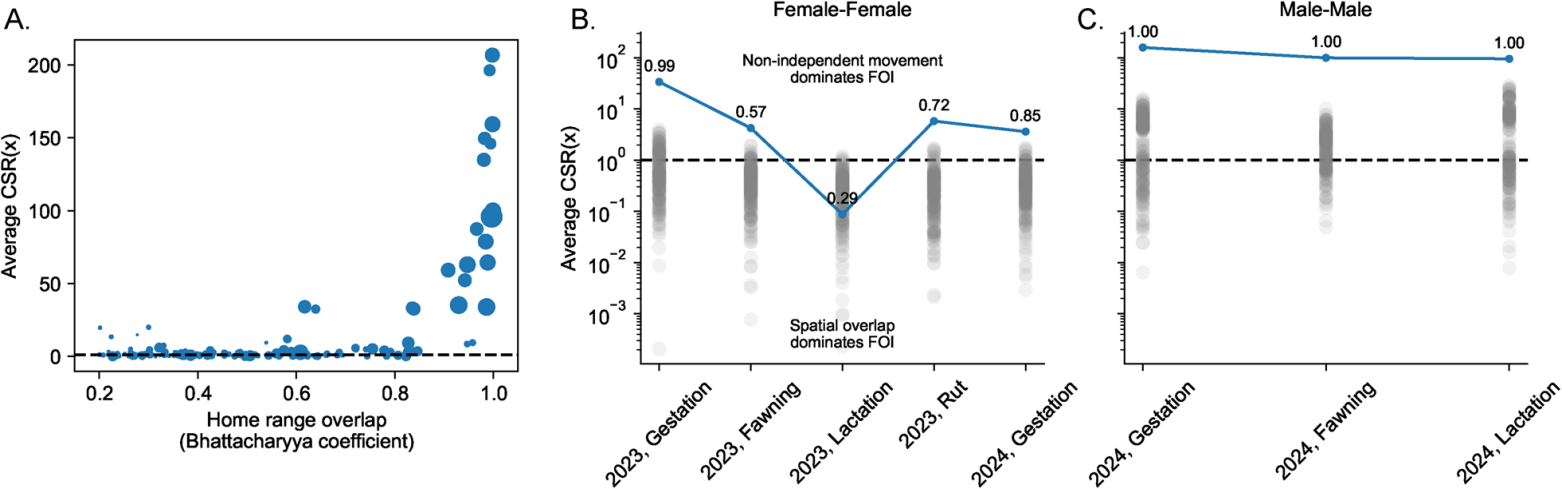
**A** The average per cell *CSR*(*x*) for 147 season by pair combinations of white-tailed deer for varying levels of home range overlap among individuals (given by the Bhattacharyya coefficient, BC; higher value means higher home range overlap). The size of points indicates the relative number of contacts (individuals within 40 m of each other) per area of overlap per unit time of collar overlap. Bigger points mean more interactions per unit area per unit time. Only pairs with $BC > 0.2$ are included on this plot. **B**, **C** Seasonal trends in the average per cell *CSR*(*x*) for a female–female pair (**B**) and a male-male pair (**C**). Blue lines give the observed average per cell *CSR*(*x*) for each pair in each season and gray points give the distribution of points from 200 temporal randomizations of host movement trajectories to remove non-independent movement [38]. If observed blue points do not overlap with the gray points, non-independent movements are contributing substantially more to transmission risk than spatial overlap. Numbers above each point give the Bhattacharyya coefficient for home range overlap for that particular pair and season

Non-independent movements substantially increased transmission risk compared to spatial overlap for the empirical movements of white-tailed deer in our system (given an area of transmission $A_x = 40\,m \times 40\,m$, Fig. 4A). There was a strong correlation between home range overlap (as calculated by the Bhattacharyya Coefficient, BC, ranging between 0 [low overlap] and 1 [high overlap]) and the degree to which non-independent movements contributed to transmission risk (Fig. 4A). In other words, if pairs of deer had high home range overlap, they also tended to be highly correlated in their space use to a degree that greatly increased transmission risk. There were instances, however, where home range overlap was high ($BC > 0.9$), but the CSR was low relative to other pairs with similar degrees of home range overlap (Fig. 4A). In these situations, deer were largely using the same space, but were not socially interacting. Conversely, there were some pairs of deer that had lower home range overlap ($BC < 0.6$), but had non-independent movements that notably affected transmission risk (Fig. 4A). In these situations, deer had strong interactions with other deer away from the core of their seasonal home range. For example, one of these pairs were two males during the gestation season that generally stayed about 1 km apart. However, over the season they had repeated bouts of interactions away from their home range centers at the northern edge of their home ranges. This pattern may represent more fluid bachelor group dynamics in male deer [40].

Focusing on two pairs of deer that had strong social interactions (a female–female pair and a male-male pair), we found that *CSR* correctly captured what we expected biologically (Fig. 4B). The transmission risk between the female–female pair was driven strongly by non-independent movements during gestation before shifting to be driven more by spatial overlap during fawning and lactation (Fig. 4B). Interestingly, while *CSR* was again augmented relative to spatial overlap following the lactation period, it remained smaller than it was previously (suggesting a possible shift in the social interactions between this pair). In contrast, the male-male pair maintained strong social connections throughout these biological seasons, and non-independent movement contributed >100 times more to transmission risk than spatial overlap. Note, we do not have data from this male-male pair during rut.

**Fig. 5:**
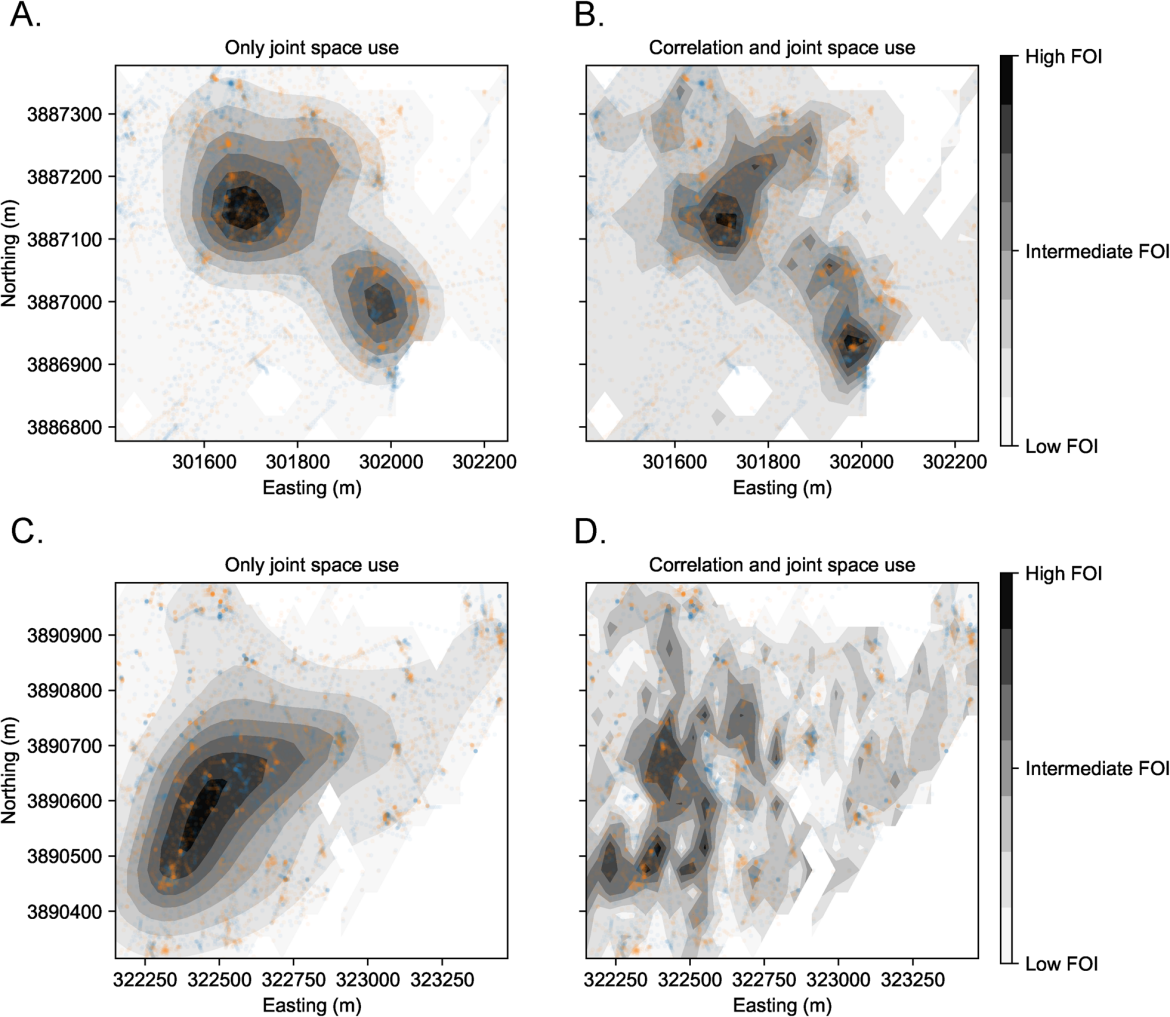
**A**, **C** A spatial map of the relative FOI due to only joint space use between two hosts. **B**, **D** A spatial map of the FOI between two hosts due to both non-independent, correlated movements and joint space use. A. and B. are a female–female pair during the gestation period and **C** and **D** are a male-male pair during the gestation period. Darker colors on the contour indicate higher relative FOI for a particular panel, but should not be compared across panels. Colored points are the observed locations of each host (one host is blue and one host is orange)

Finally, we examined whether non-independent movements reshaped the location of transmission hotspots on the landscape compared to what would be expected from joint space use and independent movements. First focusing on the female–female pair during gestation in 2023, we saw that non-independent movement drastically augmented transmission risk in areas where patterns of joint space use already predicted high transmission risk (Fig. 5A, B). As such, while there was some evidence that the locations of hotspots shifted (observed test statistic 2 standard deviations above the mean of the null, randomized distribution; $p = 0.025$ of seeing the observed change in spatial hotspots if hosts were moving independently based on a randomized KW test), the spatial structure of the transmission landscape was generally similar. In contrast, considering the male-male pair during gestation in 2024, there was a notable shift in the transmission landscape due to non-independent movement (observed test statistic 5 standard deviations above the mean of the null distribution; $p < 0.001$ of seeing the observed change in spatial hotspots if hosts were moving independently, based on a randomized KW test) – many observed areas of high transmission risk were not consistent with predictions from joint space use (Fig. 5C, D).

## Discussion

Spatial and social factors jointly contribute to transmission risk [35, 41], but we lack a rigorous way to discriminate the relative contributions of these two factors. Our new model, PMoveSTIR, disentangles the contributions of spatial overlap and non-independent movements to spatio-temporal contact and transmission risk. Using analytical results, simulations, and empirical data we show that i) non-independent movements can significantly alter transmission dynamics for short-lived pathogens, but are less important for long-lived pathogens relative to space use ii) the scale at which host social decisions are made relative to the area of pathogen transmission modulates the contributions of non-independent movements to transmission risk and iii) even weak correlations in movement between pairs on the landscape can significantly alter population-level disease dynamics of directly transmitted pathogens compared to standard assumptions of independent movement. Overall, our theory quantifies what has been largely ignored in epidemiological theory – correlation in movement can reshape epidemiological landscapes, leading to hotspots of transmission whose magnitude and location are not necessarily predictable from models of joint space use, particularly for directly transmitted pathogens.

A key motivation behind the development of movement-driven modeling of spatio-temporal risk is to more closely link empirical movement and contact data with epidemiological models. This theory provides a clear, quantitative guide to assess when fine-scale, temporally synchronous movement data are necessary for capturing disease dynamics and when coarser scale, asynchronous data focused strictly on UD estimation are sufficient. In particular, we show that non-independent movements are likely critical aspects of transmission for faster-paced pathogens, i.e. pathogens with short environmental persistence and transmission driven by short-term contacts [cf. 1, 2], such as canine distemper virus, rabies, or SARS-CoV-2. This result is broadly applicable for directly transmitted pathogens, as long as i) the area of transmission for a pathogen is small relative to the area over which hosts move and ii) social factors influencing host movement occur at similar scales as pathogen transmission. As such, empirically capturing socially driven, transmission-relevant interactions requires movement data collected on fine-temporal scales and synchronously on interacting animals. For example, in our analysis of empirical white-tailed deer infected with a hypothetical fast-paced pathogen, we found that ignoring correlation in movements and focusing only on patterns of space use could lead to i) greater than 10-fold underestimation of the magnitude of transmission experienced between individuals and ii) mis-characterization of the spatial configuration of transmission hotspots on the landscape. In contrast, we showed that non-independent movements likely have minimal effects in systems where pathogens have long persistence times (e.g., chronic wasting disease), where hosts have highly localized areas of use, and where social interactions are not responsible for bringing individuals within transmission-relevant distances. In these situations, movement data identifying coarser patterns of joint space use (e.g., GPS collars with low fix rates or spatially-explicit capture-recapture data from camera traps) are largely sufficient for understanding local transmission risk – and are often easier and cheaper to collect.

Our study repeatedly identified the potentially sizable contributions of non-independent movements to FOI. The effect of non-independent movement on spatio-temporal FOI is captured by the quantity we refer to as the pairwise correlation surface. The pairwise correlation surface reflects myriad social processes, including herding, parents with their offspring, or breeding-related interactions [42–44]. White-tailed deer female groups, for example, have high social affinity. Thus, pairs of deer with equivalent habitat overlap have substantially higher contact rates when both individuals are within the same social group [8–10, 45]. In our empirical study, the female–female pair was a mother-daughter pair. The overlap and interaction among them is consistent with the rose petal hypothesis [46], in which the home ranges of offspring radiate around the home range of their parent, and social interactions change seasonally while joint space use stays relatively similar. Because of the correlation surface between these individuals during gestation, the FOI was 33 times greater than what we expected compared to only spatial overlap [e.g., using a metric like CDE, 24]. While the importance of social interactions for contact rates has been documented previously in white-tailed deer [9, 10], the correlation surface allows us to quantify the contribution of these social interactions to the FOI, linking directly with epidemiological dynamics.

While the pairwise correlation surface is a fundamental component of spatio-temporal transmission risk, it presents three key challenges moving forward. First, our empirical analyses and analytical results assumed statistical stationarity in UDs and the correlation surface. While this is a useful simplifying assumption, spatial and social dynamics can be highly dynamic in many wildlife systems, setting up feedbacks between space use and social interactions that jointly affect UDs and correlation surfaces [18]. Ignoring these transient dynamics can lead to mis-characterization of the role of non-independent movements compared spatial overlap on transmission risk. While PMoveSTIR can be formulated in terms of transient UDs and correlation surfaces (see Appendix 3), non-parametrically estimating transient UDs at fine temporal scales with limited data points is not often feasible. A useful future direction would be to link PMoveSTIR with other recent contact theory that focuses on transient contact and transmission dynamics [but that does not consider non-independent movements, 3, 4].

Second, space use affects social interactions and social interactions affect space use [41]. The mutual feedback between these processes means that separating space use and non-independent movements is not the same thing as separating social and spatial processes. Social and spatial processes are lower-lever quantities (e.g., parameters in a mechanistic model) that lead to the emergent patterns of UDs and correlation surfaces. To illustrate this, consider our messy follow-the-leader model. The social process in this model is the probability of following the leader and the spatial process is the selection of space conditional on not following the leader. The emergent correlation surface is a function of the social process $p_f$ and spatial process of a dominant individual randomly choosing a cell to “use”. Thus, the correlation surface should be conceptualized as an interaction between spatial and social processes, and is zero when hosts are moving independently. An important next step is to link PMoveSTIR theory to step-selection models [e.g., 44] to further understand how social and spatial processes map to joint UDs and pairwise correlation surfaces – the emergent quantities that ultimately matter for transmission risk.

Finally, estimating the empirical correlation surface can be challenging. While even small levels of correlation in host space use can significantly influence spatio-temporal infection risk, precisely estimating correlation surfaces with sufficient precision from a finite amount of movement data can be difficult, particularly for small areas of transmission. We showed that randomization approaches can partially mitigate this issue [e.g., 38], allowing us to establish baseline expectations of correlation contributions to FOI based on a null model of independent movement. Moving forward, a viable strategy might be to generate correlation surfaces from lower-level movement models fit to observed movement data [e.g., 22].

A broad goal of PMoveSTIR is to help scale from individual movements to population-level epidemiological dynamics. There are three central components to the total force of infection felt by an individual on the landscape: i) how many hosts are on the landscape ii) how transmission occurs through shared space use and iii) how transmission occurs through non-independent movement. The first component requires estimates of host density in a population that might occur in conjunction with the collection of individual movement data. The latter two components depend strongly on individual movements collected from tracking data. Once host density and individual movement data are collected, the key challenge is scaling up individual movements from a subset of tracked individuals to the entire population. Individual movement data is useful because to estimate FOI, PMoveSTIR needs to know a) where individuals are establishing home ranges on the landscape [e.g., second-order selection 47] – which can be done (at least partially) from individual movement data, b) how individuals are using space within their home range [e.g., third-order selection 47], – which can be estimated from UDs or step-selection analysis [22], and c) how correlated space use is between pairs of individuals across their area of use – which we can estimate using the approaches developed in this study. By using methods like step-selection analysis with individual-level random effects [48] and empirical bootstrapping one could then assign the above-mentioned components to each individual in an *in silico* population, use PMoveSTIR to compute spatially-explicit FOI between all pairs of individuals, and construct a movement-based contact network to explore disease dynamics in the population. This is a coarse description of how we might scale-up from individual movement data to population-level disease dynamics. Many key sampling and biological challenges remain to be addressed, such as tracking a sufficient number of individuals to reliably bootstrap patterns of space use and non-independent movements to the population-level, incorporating realistic group size dynamics (perhaps through independent estimates of group size distributions), and accounting for seasonal changes in space use and social interactions. However, by partitioning FOI into components that are directly estimable from common movement data and linking tightly with epidemiological theory, PMoveSTIR provides a useful piece in the puzzle of how to scale-up from samples of individual movements to population-level disease dynamics.

## Conclusion

A goal at the interface of movement and disease ecology is to create an epidemiological risk landscape independent of geographical location [2, 49], which could be achieved by linking movement data and inferred contact with underlying environmental factors. Establishing these contact-environment links could enable predictions of transmission risk on landscapes that extend beyond observed movement trajectories and collared individuals. For example, contact-environment links would allow for forecasting future disease dynamics in the study population or quantifying transmission risk for other populations in similar environments. Our model provides a generalizable theoretical foundation to perform similar analyses across different host-pathogen systems, and it can be integrated with any UD estimation method [22, 27, 49, 50]. In addition to movement covariates, PMoveSTIR can also incorporate spatially and temporally heterogeneous epidemiological parameters, for example pathogen survival rates that vary between habitats and seasons [51], or spatially localized shedding [52]. However, our results show that an essential feature of any approach that seeks to predict landscape-level epidemiological risk beyond observed movement trajectories will be rigorous estimation of the pairwise correlation surface. Our analyses emphasize the importance of this surface, showing that correlations at a local scale can restructure transmission landscapes and may contribute to the growing empirical recognition that fine-scale, localized transmission hotspots are present in many empirical host-pathogen systems [11, 12]. Whether these localized transmission hotspots are predictable *a priori* remains to be seen, but ignoring non-independent movements can make the task orders of magnitude more difficult for some pathogens.

## Supplementary Information

Supplementary file 1.

## Data Availability

Code and data necessary to replicate all results in this manuscript are provided at https://github.com/mqwilber/pmovestir.

## Abbreviations

UD: Utilization distribution
FOI: Force of infection
CDE: Conditional distribution of encounters
OU: Ornstein-uhlenbeck
CSR: Correlation-spatial overlap ratio
KDE: Kernel density estimates
KW: Kruskal–Wallis
BC: Bhattacharyya coefficient
