# Supplementary material for: How do non-independent host movements affect spatio-temporal disease dynamics? Partitioning the contributions of spatial overlap and correlated movements to transmission risk
Source: Mov Ecol. 2025 Feb 26;13:11. doi: 10.1186/s40462-025-00539-4 (PMC11866778; doi:10.1186/s40462-025-00539-4)
Supplement: Supplementary file 1 [file 40462_2025_539_MOESM1_ESM.pdf]

1 Derivation of MoveSTIR

The text below is taken directly from [1] to orient readers.

MoveSTIR begins from a simple compartmental host-parasite model that tracks **S**usceptible and **I**nfected host density and the density of the **P**athogen in the environment. We make the following assumptions: i) the force of infection (FOI) experienced by a susceptible host is a linear function of pathogen density in the environment βP, where β is the transmission rate that combines rates of acquisition and contact, ii) infected hosts deposit pathogen at rate λ, iii) the pathogen decays in the environment at rate ν, iv) the pathogen is well-mixed in the area where contact and acquisition occur, and v) the infection process does not substantially deplete the pathogen in the environment [2; 3]. These assumptions provide a reasonable starting point for MoveSTIR but can be readily adjusted to account for non-linear FOI or extended to account for states such as **E**xposed and **R**ecovered. For simplicity, we also assume that **I**nfected hosts recover at rate γ and are immediately **S**usceptible.

The following equations describe the infection dynamics in a host population

$$\begin{aligned}\frac{dS}{dt} &= -\beta PS + \gamma I \\ \frac{dI}{dt} &= \beta PS - \gamma I \\ \frac{dP}{dt} &= \lambda I - \nu P\end{aligned} \tag{S1}$$

where we initially assume a constant population size of $S + I = H$ and no births or deaths. Equation S1 is applicable to both microparasites (e.g., bacteria and viruses) or macroparasites (e.g., helminths) with a simple life cycle [e.g., 3].

We can equivalently express equation S1 as a renewal equation [4], namely

$$\begin{aligned}\frac{dS(t)}{dt} &= -\beta P(t)S(t) + \gamma I(t) \\ \frac{dI(t)}{dt} &= \beta P(t)S(t) - \gamma I(t) \\ P(t) &= P_0(t) + \int_0^t \lambda I(u) e^{-\nu(t-u)} du\end{aligned} \tag{S2}$$

In equation S2, $I(u)$ gives the density of infected individuals at some previous time u, $u < t$. The function $e^{-\nu(t-u)}$ defines the pathogen survival function in the environment, assuming that the pathogen decays at a constant rate ν. We could readily replace $e^{-\nu(t-u)}$ with any survival function reflective of the pathogen of interest $\Theta(t-u)$. The parameter $P_0(t)$ is the density of pathogen present at time 0 that are still present at time t.

We can then substitute in the expression of $P(t)$ to re-write $\frac{dI(t)}{dt}$ as a function of $I(u)$

$$\frac{dI(t)}{dt} = S(t)\beta \int_0^t \lambda I(u)e^{-\nu(t-u)}du - \gamma I(t) \tag{S3}$$

where we assume $P_0(t) = 0$.

The function $h(t) = \beta \int_0^t \lambda I(u)e^{-\nu(t-u)}du$ is the per capita FOI at time t. The FOI $h(t)$ is a rate with units time^{-1}. As such, $h(t)$ defines the FOI felt by an individual at a given moment after accounting for the time-dependent accumulation and decay of all pathogens previously deposited by infected hosts. Importantly, $h(t)$ lets deposition rate (λ) and contact formation and acquisition of the pathogen (both encapsulated in β) vary independently, and allows for a continuum between direct contact (when $u \approx t$) and indirect contact (when $u < t$). However, equation S3 does not i) clearly separate contact formation and pathogen acquisition, ii) explicitly account for contact duration, or iii) account for directional differences in transmission risk due to the order of when individuals visit locations. We extended equation S3 to consider directional interactions occurring at the individual level.

A pairwise view of the force of infection (FOI)

Consider a single individual i moving through space. At each moment, the individual experiences a FOI dependent upon the full history of infected individuals that previously or presently share its current location. Let $I(u, x)$ be the number of infected hosts in location x at time u. If there are $H - 1$ other hosts in the population, we can write $I(u, x) = \sum_{j=1}^{H-1} \delta_{x_j(u)}(x)\delta_{I_j(u)}(I)$. The function $\delta_{x_j(u)}(x)$ is an indicator function that is defined as

$$\delta_{x_j(u)}(x) = \begin{cases} 1 & \text{if the location of host } j \text{ at time } u \text{ is } x \text{ (i.e., } x_j(u) = x) \\ 0 & \text{otherwise} \end{cases}$$

Similarly, $\delta_{I_j(u)}(I)$ is the indicator function

$$\delta_{I_j(u)}(I) = \begin{cases} 1 & \text{if host } j \text{ is infected at time } u \text{ (i.e., } I_j(u) = I) \\ 0 & \text{otherwise} \end{cases}$$

Taken together, this means that host j at past time u only gets "counted" toward the FOI experienced by focal host i at time t if they are infected and shedding pathogen ($I_j(u) = I$) at time u and in the same location x ($x_j(u) = x$).

We can then update $h(t)$ to consider the contributions from other individual hosts to the FOI felt by a focal host i at time t in location x.

$$
\begin{aligned}
h_i(t,x) &= \beta' \int_0^t \sum_{j=1}^{H-1} \lambda \delta_{x_j(u)}(x) \delta_{I_j(u)}(I) \Theta(t-u) du \\
&= \underbrace{\sum_{j=1}^{H-1}}_{\text{Sum over individuals}} \int_0^t \underbrace{\beta'}_{\text{Acquisition}} \underbrace{\delta_{x_j(u)}(x)}_{\text{Contact}} \underbrace{\lambda \delta_{I_j(u)}(I)}_{\text{Deposition}} \underbrace{\Theta(t-u)}_{\text{Pathogen decay}} du
\end{aligned}
\tag{S4}
$$

where β' is now an acquisition rate (i.e., an uptake rate times per pathogen probability of infection) as we have conditioned on contact with the term $\delta_{x_j(u)}(x)$. Consider a single term in the summation, $h_{i \leftarrow j}(t,x) = \int_0^t \beta' \delta_{x_j(u)}(x) \lambda \delta_{I_j(u)}(I) \Theta(t-u) du$. We can define this term as: the FOI felt by individual $i \neq j$ at time t in location x due to individual j's previous infection history in location x, up to time t. This quantity is a rate with units time^{-1} and encapsulates pathogen acquisition, contact formation, pathogen deposition, and direct and indirect transmission. Contact duration is explicitly accounted for by the integral over $\delta_{x_j(u)}(x)$, which specifies how long host i is in contact with host j in the past (indirect) or present (direct). Finally, we can more explicitly account for the area of location x by re-writing $\beta' \delta_{x_j(u)}(x)$ as $\tilde{\beta} \Phi(x_j(u), x)$ [5; 6], where $\tilde{\beta}$ has units $\frac{\text{area units}}{\text{time}}$ (e.g., $\frac{m^2}{\text{hour}}$). The function $\Phi(x_j(u), x)$ is the contact function and is a probability density function that integrates to one over the spatial domain of interest with units 1/area units [1].

2 PMoveSTIR in continuous space

In the main text, we derive PMoveSTIR assuming that hosts are contacting each other within some area of transmission A_x, which we can conceptually think about as a grid cell on a gridded landscape. While this conceptually simplifies the problem, it is more general to consider the case of continuous space where we define contact as potentially happening when present or past host j is (was) within some distance r of host i at its present location [1]. The output we want from this alternative version of PMoveSTIR is the function $\hat{h^*}(x,t)$, which is the force of infection *per unit area* at point x on the landscape (e.g., $\hat{h^*}(x,t)$ might have units day^{-1}m^{-2}). Integrating this function over different areas will yield estimates of force of infection felt by host i from host j for any area of interest on the landscape.

Let's start with the situation where a host i is occupying some circular area $A_{x,\rho}$ where x is the center of the area and ρ is the radius of the area. A contact can occur when host j (past or present) is in the area $A_{x,\rho+r}$ where r is our epidemilogically relevant contact distance and $r >> \rho$. The force of infection felt by host i from host j as time t in area $A_{x,\rho}$ is given by

$$
h_{i \leftarrow j}(t, A_{x,\rho}) = \int_{-\infty}^{t} \beta' \lambda \delta'_{x_i(t)}(A_{x,\rho}) \delta'_{x_j(u)}(A_{x,\rho+r}) \Theta(t-u) du. \tag{S5}
$$

where, consistent with the main text, $\delta'_{x_i(t)}(A_{x,\rho})$ is a Bernoulli random variable that determines whether or not host i is located in area $A_{x,\rho}$ at time t and $\delta'_{x_j(u)}(A_{x,\rho+r})$ is a Bernoulli random variable that determines whether or not host j is in area $A_{x,\rho+r}$ at time u. The variables $x_i(t)$ and $x_j(u)$ indicate the locations of host i and j at time t and u, respectively. The parameter $\beta' = \frac{\tilde{\beta}}{A_{x,\rho+r}}$ and indicates our assumption that encounters are equally likely within an area $A_{x,\rho+r}$. The parameter λ is the pathogen shedding rate of host j and ν is the pathogen decay rate once deposited in the environment. As in the main text, we are computing maximum transmission risk and assuming that host j is always infected at any time u.

We can envision simulating many different movement trajectories for host i and j and take the expectation of $h_{i\leftarrow j}(t, A_{x,\rho})$. We obtain

$$h^*_{i\leftarrow j}(t, A_{x,\rho}) = \int_{-\infty}^{t} \beta' \lambda E[\delta'_{x_i(t)}(A_{x,\rho})\delta'_{x_j(u)}(A_{x,\rho+r})]\Theta(t-u)du. \tag{S6}$$

We can rewrite equation S6 as

$$h^*_{i\leftarrow j}(t, A_{x,\rho}) = \frac{\tilde{\beta}}{A_{x,\rho+r}}\lambda \int_{-\infty}^{t} [p_i(A_{x,\rho}, t)p_j(A_{x,\rho+r}, u) + Cov(\delta'_{x_i(t)}(A_{x,\rho}), \delta'_{x_j(u)}(A_{x,\rho+r}))]\Theta(t-u)du, \tag{S7}$$

where the force of infection felt by host i from host j is the related to the utilization distributions of the two hosts and their covariance within an area (in actuality, two nested areas).

For simplicity, consider hosts moving independently. In this case, we can write

$$h^*_{i\leftarrow j}(t, A_{x,\rho}) = \beta' \lambda \int_{-\infty}^{t} [p_i(A_{x,\rho}, t)p_j(A_{x,\rho+r}, u)]\Theta(t-u)du \tag{S8}$$

where $p_i(A_{x,\rho}, t)$ is the probability of host i being in area $A_{x,\rho}$ at time t and $p_j(A_{x,\rho+r}, u)$ is the probability of host j being in area $A_{x,\rho+r}$ at time u.

Now we want to calculate $h^*_{i\leftarrow j}(t, A_{x,\rho})$ in the limit as $\rho \to 0$. For equation S8, we can divide both sides by $A_{x,\rho}$ and take the limit as $\rho \to 0$ (such that $A_{x,\rho} \to 0$). Doing this we obtain

$$\hat{h}^*_{i\leftarrow j}(t, x) = \beta' \lambda \int_{-\infty}^{t} [f_i(x, t)p_j(A_{x,r}, u)]\Theta(t-u)du = \beta' \lambda \int_{-\infty}^{t} [f_i(x, t) \int_{\chi \in A_{x,r}} f_j(\chi, u)d\chi]\Theta(t-u)du \tag{S9}$$

where $f_i(x, t)$ and $f_j(x, t)$ are the probability density functions of space use for host i and host j, respectively. The term $\int_{\chi \in A_{x,r}}$ indicates that we are integrating over all spatial locations χ within the area $A_{x,r}$. Note that the units on $f_i(x, t)$ or $f_j(x, t)$ are per area, such that the force of infection $\hat{h}^*_{i\leftarrow j}(t, x)$ has units per

time per area as opposed to $h^*_{i\leftarrow j}(t, A_{x,\rho})$ which has units per time. Conceptually, for $h^*_{i\leftarrow j}(t, A_{x,\rho})$ we have already integrated over area so we cancel out the per area units.

Assuming a stationary process and that $\Theta(t-u) = e^{-\nu(t-u)}$, we can write equation S9 as

$$\hat{h}^*_{i\leftarrow j}(t, x) = \frac{\beta'\lambda}{\nu}[f_i(x)\int_{\chi\in A_{x,r}} f_j(\chi)d\chi] \tag{S10}$$

Furthermore, if we assume that the space use of host j is relatively uniform within the transmission area $A_{x,r}$ we can simplify to

$$\hat{h}^*_{i\leftarrow j}(t, x) = \frac{\beta'\lambda}{\nu}[f_i(x)f_j(x)\pi r^2] \tag{S11}$$

Remembering that $\beta' = \tilde{\beta}/A_{x,r} = \tilde{\beta}/\pi r^2$, we get

$$\hat{h}^*_{i\leftarrow j}(t, x) = \frac{\tilde{\beta}\lambda}{\nu}[f_i(x)f_j(x)] \tag{S12}$$

Integrating $\hat{h}^*_{i\leftarrow j}(t, x)$ over some area of interest centered at x would yield $h^*_{i\leftarrow j}(t, A_{x,d}) = \frac{\tilde{\beta}\lambda}{\nu}\int_{\chi\in A_{x,d}}[f_i(\chi)f_j(\chi)d\chi]$. This is reminiscent of the equation 17 in [6] where a limiting case of the mean encounter rate of two individuals moving according to an Ornstein-Uhlenbeck movement process is proportional to the inner product of their utilization distributions.

Including correlation in movement into this formulation of PMoveSTIR is more theoretically and empirically challenging, and we leave this task for a later paper.

3 Deriving PMoveSTIR given an assumption of statistical stationarity

We now derive the PMoveSTIR model from equation 1 in the main text. First, we simplify equation 1 in the main text by assuming that the depositing host is always infectious and shedding pathogen at a constant rate. This assumption is equivalent to building a contact network and also represents the structural form of FOI needed to compute pathogen invasion thresholds such as R_0 [see 1].

Second, we consider probabilistic space use (i.e., we know where an individual is at a given time and location with some probability) and re-write equation 1 in the main text as

$$h_{i\leftarrow j}(t, x) = \int_{-\infty}^{t} \beta'\lambda\delta'_{x_i(t)}(x)\delta'_{x_j(u)}(x)\Theta(t-u)du \tag{S13}$$

where $\delta'_{x_i(t)}(x)$ is a random variable that specifies whether host i is in location x at time t (defined equivalently for j). This means that $h_{i\leftarrow j}(t,x)$ is also a random variable, and we can express its expected value as

$$h^*_{i\leftarrow j}(t,x) := E[h_{i\leftarrow j}(t,x)] = \int_{-\infty}^{t} \beta'\lambda E[\delta'_{x_i(t)}(x)\delta'_{x_j(u)}(x)]\Theta(t-u)du. \tag{S14}$$

Interpreting this expectation, we are asking: if we simulated some movement process thousands of times, what is the probability that host i is in location x at time t, and host j was in x at a previous time u? While this is a simple mathematical extension to the MoveSTIR equation S4, it is a significant conceptual advance as it provides the mathematical structure necessary to link FOI directly with UDs and partition the contributions of spatial overlap and non-independent movements on FOI, as we describe next.

For two random variables Y and Z, $E[YZ] = E[Y]E[Z] + Cov(Y,Z)$. We can therefore write equation S14 as

$$\begin{aligned} h^*_{i\leftarrow j}(t,x) &= \int_{-\infty}^{t} \frac{\tilde{\beta}}{A_x}\lambda E[\delta'_{x_i(t)}(x)\delta'_{x_j(u)}(x)]\Theta(t-u)du \\ &= \frac{\tilde{\beta}}{A_x}\lambda \int_{-\infty}^{t} [E[\delta'_{x_i(t)}(x)]E[\delta'_{x_j(u)}(x)] + Cov(\delta'_{x_i(t)}(x), \delta'_{x_j(u)}(x))]\Theta(t-u)du \\ &= \frac{\tilde{\beta}}{A_x}\lambda \int_{-\infty}^{t} [p_i(x,t)p_j(x,u) + Cov(\delta'_{x_i(t)}(x), \delta'_{x_j(u)}(x))]\Theta(t-u)du \end{aligned} \tag{S15}$$

where we use the fact that the expectation of an indicator variable is a probability [7]. The terms $p_i(x,t)$ and $p_j(x,u)$ give the probabilities that host i and j are in location x at times t and u, respectively, and can also be written as $p_i(x,t) = \int_{\chi\in A_x} f_i(\chi,t)d\chi$ where $f_i(\chi,t)$ is the probability density of host i using the point k at time t and the integral is over the area of transmission A_x (defined equivalently for host j). Thus, we have obtained an equation that links the transient utilization distributions $f_i(\chi,t)$ and $f_j(\chi,u)$ with the spatio-temporal FOI. The parameters $\tilde{\beta}$ and λ are the acquisition and deposition rates respectively, and A_x is the area of transmission at location x. $Cov(\delta'_{x_i(t)}(x), \delta'_{x_j(u)}(x))$ gives the covariance in how host i and j at time t and u are using area x. Finally, $\Theta(t-u)$ gives the survival probability of a pathogen at time t that was deposited at time u.

Equation S15 accounts for heterogeneity in space and time, but the model can consider other scenarios, such as uniform space use or UDs that are stationary in time. In terms of the novelty of equation S15, we note that [1] in Appendix 9 considered a special case of PMoveSTIR looking at home-range overlap, but they did not link this equation to UDs and ignored covariance in movement.

To derive equation 2 in the main text that assumes stationarity in utilization distributions, we start with equation S15. If we assume a stationary utilization distribution, the time indexes on $p_i(x,t)$ and $p_j(x,u)$ are irrelevant – the logic here is that, by definition, the mean of a stationary distribution is independent of time so $p_i(x,t) = p_i(x)$ and $p_j(x,u) = p_j(x)$. Therefore, we can write

$$h^*_{i\leftarrow j}(t,x) = \frac{\tilde{\beta}}{A_x}\lambda\left[p_i(x)p_j(x)\int_{-\infty}^{t}\Theta(t-u)du + \int_{-\infty}^{t} Cov(\delta'_{x_i(t)}(x), \delta'_{x_j(u)}(x))\Theta(t-u)du\right].$$

In addition, given stationarity, $h^*_{i\leftarrow j}(t,x)$ does not depend on time, such that

$$h^*_{i\leftarrow j}(x) = \frac{\tilde{\beta}}{A_x}\lambda\left[p_i(x)p_j(x)\int_{-\infty}^{t}\Theta(t-u)du + \int_{-\infty}^{t} Cov(\delta'_{x_i(t)}(x), \delta'_{x_j(u)}(x))\Theta(t-u)du\right].$$

Moreover, given stationarity, we know that $Cov(\delta'_{x_i(t)}(x), \delta'_{x_j(u)}(x))$ just depends on the time lag $s = t-u$ and not specific time stamps. Thus, we can write

$$h^*_{i\leftarrow j}(x) = \frac{\tilde{\beta}}{A_x}\lambda\left[p_i(x)p_j(x)\int_{0}^{\infty}\Theta(s)ds + \int_{0}^{\infty} Cov(\delta'_{x_i(k)}(x), \delta'_{x_j(k-s)}(x))\Theta(s)ds\right]. \quad \text{(S16)}$$

The term $Cov(\delta'_{x_i(k)}(x), \delta'_{x_j(k-s)}(x))$ is the (lagged) covariance between the occupancy random variables $\delta'_{x_i(k)}(x)$ and $\delta'_{x_j(k-s)}(x)$ for any time k where the time lag is s. Given the assumption of statistical stationarity, the covariance between the occupancy random variables of host i and host j does not depend on absolute time k or $k-s$, but just the time lag s.

We can then write the term $Cov(\delta'_{x_i(k)}(x), \delta'_{x_j(k-s)}(x))$ as

$$Cov(\delta'_{x_i(k)}(x), \delta'_{x_j(k-s)}(x)) = SD(\delta'_{x_i(k)}(x))SD(\delta'_{x_j(k-s)}(x))Cor(\delta'_{x_i(k)}(x), \delta'_{x_j(k-s)}(x)) \quad \text{(S17)}$$

where SD is the standard deviation of the random variables of occupancy of location x for host i and j. Since the marginal distribution of host i's occupancy (marginalizing over host j's occupancy) follows a Bernoulli distribution (i.e., host i can only be in or not in location x with some probability), then $SD(\delta'_{x_i(k)}(x)) = \sqrt{p_i(x,k)(1-p_i(x,k))}$. Because we are assuming stationarity (specifically, wide-sense stationarity), $p_i(x,k) = p_i(x)$ and we get $\sqrt{p_i(x)(1-p_i(x))} = \sigma_i(x)$. The same holds for host j. We can then write

$$h^*_{i\leftarrow j}(x) = \frac{\tilde{\beta}}{A_x}\lambda\left[p_i(x)p_j(x)\int_{0}^{\infty}\Theta(s)ds + \sigma_i(x)\sigma_j(x)\int_{0}^{\infty} Cor(\delta'_{x_i(k)}(x), \delta'_{x_j(k-s)}(x))\Theta(s)ds\right]. \quad \text{(S18)}$$

4 Deriving general PMoveSTIR results when space use is uniform and transmission is from direct contact

Here, we derive the general PMoveSTIR results from an assumption that space use is uniform and transmission is primarily from direct contact. First, we can re-write equation S18 as

$$h^*_{i\leftarrow j}(x) = \beta'\lambda[\underbrace{\frac{A_x}{A_{tot}}\frac{A_x}{A_{tot}}\int_0^\infty \Theta(s)ds}_{\substack{\text{FOI contribution from}\\ \text{spatial overlap}}} + \underbrace{\frac{A_x}{A_{tot}}(1-\frac{A_x}{A_{tot}})\int_0^\infty \rho(x,s)\Theta(s)ds}_{\substack{\text{FOI contribution from}\\ \text{non-independent movement}}}]. \tag{S19}$$

where we substituted $p_i(x) = p_j(x) = \frac{A_x}{A_{tot}}$ because both hosts are using space uniformly. Moreover, $\sigma_i(x)\sigma_j(x) = \sqrt{\frac{A_x}{A_{tot}}(1-\frac{A_x}{A_{tot}})}\sqrt{\frac{A_x}{A_{tot}}(1-\frac{A_x}{A_{tot}})} = \frac{A_x}{A_{tot}}(1-\frac{A_x}{A_{tot}})$. Finally, as defined in the main text, $Cor(\delta'_{x_i(k)}(x), \delta'_{x_j(k-s)}(x)) = \rho(x,s)$.

Further, we assume that the pathogen survival function $\Theta(s)$ is a step function with a survival probability of one when lag $s \leq T$ and zero when $s > T$. With these assumptions, we can write equation S19 as

$$h^*_{i\leftarrow j}(x) = \beta'\lambda\left[\frac{A_x}{A_{tot}}\frac{A_x}{A_{tot}}T + \frac{A_x}{A_{tot}}(1-\frac{A_x}{A_{tot}})\int_0^T \rho(x,s)ds\right], \tag{S20}$$

The correlation term $\rho(x,s)$ will be exactly $\rho(x,s=0)$ when lag $s=0$ (in words, $\rho(x,s=0)$ is the correlation in two hosts' space use with a time lag of 0) and near $\rho(x,s=0)$ when lag s is near zero. When pathogens are strictly directly transmitted, T is small relative to the duration of time hosts spend in an area of transmission and we can reasonably approximate $\rho(x,s) = \rho(x,s=0)$ from 0 to T (assuming $\rho(x,s)$ is continuous in s). We can then write equation S20 as

$$h^*_{i\leftarrow j}(x) = \beta'\lambda T\left[\underbrace{\frac{A_x}{A_{tot}}\frac{A_x}{A_{tot}}}_{\substack{\text{Contribution due}\\ \text{to spatial overlap}}} + \underbrace{\frac{A_x}{A_{tot}}(1-\frac{A_x}{A_{tot}})\rho(x,s=0)}_{\substack{\text{Contribution due}\\ \text{to non-independent movement}}}\right]. \tag{S21}$$

which is the equation we provide in the main text.

5 Deriving analytical results for the messy follow-the-leader model

In this section, we derive the analytical results for the messy follow-the-leader model discussed in the main text. Our goal is to derive the equation

$$\rho_{messy}(x, s=0) = \frac{p_f(N_h - 1)}{(n_p N_h - 1)(N_h(n_p - p_f) + p_f)} \tag{S22}$$

that describes the lag 0 correlation within the area of transmission ($1/n_p$) between two hosts in the follow-the-leader model. The parameter N_h describes the number of patches on the landscape between which hosts can move, each patch is composed of n_p areas of transmission that hosts can co-occupy (think about these as grids within a patch), and p_f is the probability of hosts following the leader as they move over a time step. Dominant hosts always move randomly within and between patches. If a sub-dominant host does not follow the dominant host, they also move randomly.

To calculate the correlation in space use for host i and j in any location where transmission can occur x on the landscape we need to calculate $Cor(i \text{ in } x, j \text{ in } x) = \frac{Cov(i \text{ in } x, j \text{ in } x)}{SD(i \text{ in } x)SD(j \text{ in } x)}$. Here, we use the notation i in x to refer to the random 0/1 variable of host i being in location x or not (same for host j). We know that the probability of a host being in any particular area of transmission x is $[i \text{ in } x] = \frac{1}{N_h n_p}$ (equivalently for host j). Note that we use the notation '[]' to refer to a probability statement [e.g., in 8]. Since i in x is a random Bernoulli variable, $SD(i \text{ in } x) = \sqrt{\frac{1}{N_h n_p}(1 - \frac{1}{N_h n_p})}$. Further, we know that

$$\begin{aligned} Cov(i \text{ in } x, j \text{ in } x) &= E[i \text{ in } x, j \text{ in } x] - E[i \text{ in } x]E[j \text{ in } x] \\ &= [i \text{ in } x | j \text{ in } x][j \text{ in } x] - [i \text{ in } x][j \text{ in } x] \\ &= [i \text{ in } x | j \text{ in } x]\frac{1}{N_h n_p} - \frac{1}{N_h n_p}\frac{1}{N_h n_p} \end{aligned} \tag{S23}$$

To solve for $[i \text{ in } x | j \text{ in } x]$, we first need to think about the probability of host i and j being together anywhere on the landscape – [together]. We can write

$$[\text{together}] = \sum_{x \in \text{All areas of transmission}} [i \text{ in } x | j \text{ in } x][j \text{ in } x]$$

where an area of transmission is any location x of area $1/n_p$ within a patch. Since hosts are using areas of transmission randomly (note that this is true even though their space use is correlated through following the leader), $[i \text{ in } x | j \text{ in } x][j \text{ in } x]$ is the same for all x. We can then write

$$\begin{aligned} [\text{together}] &= N_h n_p [i \text{ in } x | j \text{ in } x][j \text{ in } x] \\ &= N_h n_p [i \text{ in } x | j \text{ in } x]\frac{1}{N_h n_p} \\ &= [i \text{ in } x | j \text{ in } x] \end{aligned} \tag{S24}$$

So the probability of being together in the same area of transmission anywhere on the landscape ([together])

is the same as the conditional probability of host i using an area of transmission x given host j is using that area. Thus, calculating [together] will give us [i in $x|j$ in x].

We can calculate [together] by thinking about the messy follow-the-leader model as a discrete-time, discrete-state Markov chain. In particular, consider two states that two hosts can be in at any time – Together (T), and Not together (NT). In each time step, we can write down the transition matrix $\mathbf{A}$ (a column-wise stochastic matrix) of moving between these two states given the messy follow-the-leader model as (note the letters above and to the left of the transition matrix just indicate the states for helpful reference)

$$\mathbf{A} = \begin{array}{ccc} & T & NT \\ T & p_f \frac{1}{n_p}\frac{1}{n_p} n_p + (1-p_f)\frac{1}{n_p}\frac{1}{n_p}\frac{1}{N_h}\frac{1}{N_h} N_h n_p & \frac{1}{n_p}\frac{1}{n_p}\frac{1}{N_h}\frac{1}{N_h} N_h n_p \\ NT & p_f(1-\frac{1}{n_p}\frac{1}{n_p} n_p) + (1-p_f)(1-\frac{1}{n_p}\frac{1}{n_p}\frac{1}{N_h}\frac{1}{N_h} N_h n_p) & (1-\frac{1}{n_p}\frac{1}{n_p}\frac{1}{N_h}\frac{1}{N_h} N_h n_p) \end{array} \qquad \text{(S25)}$$

Let us interpret the $T \to T$ transition probability. Consider two hosts together in the same area of transmission at time t. The probability that they are still together in some area of transmission in time $t+1$ is given by answering two questions: i) does the subordinate individual follow the leader (p_f) and, if so, do the hosts end up in the same area of transmission within the patch that they move to ($p_f \frac{1}{n_p}\frac{1}{n_p} n_p$)? and ii) does the subordinate individual not follow the leader ($1-p_f$), but just through random movement the two hosts move to the same patch and end up in the same area of transmission ($(1-p_f)\frac{1}{n_p}\frac{1}{n_p}\frac{1}{N_h}\frac{1}{N_h} N_h n_p$)? The other transition probabilities are defined using similar logic.

Using $\mathbf{A}$, we can now calculate the long-run stationary probability of the two hosts being together by obtaining the right eigenvector of $\mathbf{A}$ with the corresponding eigenvalue of 1. Doing this, we get the column vector (normalized to sum to 1)

$$\begin{bmatrix} \frac{1}{N_h(n_p-p_f)+p_f} \\ 1-\frac{1}{N_h(n_p-p_f)+p_f} \end{bmatrix},$$

where the stationary probability of being together somewhere in an area of transmission on the landscape is [together] $=$ [i in $x|j$ in x] $= \frac{1}{N_h(n_p-p_f)+p_f}$.

Combining everything, we get

$$
\begin{aligned}
Cor(i \text{ in } x, j \text{ in } x) &= \frac{Cov(i \text{ in } x, j \text{ in } x)}{SD(i \text{ in } x)SD(j \text{ in } x)} \\
&= \frac{[\text{together}]\frac{1}{N_h n_p} - \frac{1}{N_h n_p}\frac{1}{N_h n_p}}{\frac{1}{N_h n_p}(1 - \frac{1}{N_h n_p})} \\
&= \frac{(\frac{1}{N_h(n_p - p_f) + p_f})\frac{1}{N_h n_p} - \frac{1}{N_h n_p}\frac{1}{N_h n_p}}{\frac{1}{N_h n_p}(1 - \frac{1}{N_h n_p})} \\
&= \frac{p_f(N_h - 1)}{(n_p N_h - 1)(N_h(n_p - p_f) + p_f)} \\
&= \rho_{messy}(x, s = 0).
\end{aligned}
\tag{S26}
$$

6 Scaling up to the population-level for the follow-the-leader model

Here we show how given the messy follow-the-leader model described in the main text, we can derive an explicit equation for the basic reproductive number R_0 that links correlation in movement can directly to population-level disease dynamics.

We will start by writing down a discrete-time, individual-level **S**usceptible-**I**nfected-**R**ecovered model where we are tracking the probabilities that host i is in state S, I, or R – $p_{i,S}(t)$, $p_{i,I}(t)$, and $p_{i,R}(t)$ respectively. We assume there are H total individuals in the population and let $\mathbf{H}_{-i}$ be the set of all individuals, excluding the ith individual. Just as in the follow-the-leader model described in the main text and Appendix 5, we assume individuals are moving across a gridded landscape with $N_h n_p$ total grids/areas of transmission where infection can occur. We do not track exactly where individuals are on the landscape, just their infection status.

We can think about the messy follow-the-leader model as a network model where individuals are nodes and edges are defined by the average force of infection between individuals felt by their interactions over the entire landscape. Following [9] and [10], a non-linear, mean-field approximation of the individual probabilities of being Susceptible, Infected, or Recovered is

$$
\begin{aligned}
p_{i,S}(t + dt) &= p_{i,S}(t) \prod_{j \in \mathbf{H}_{-i}} (1 - \text{Prob. } i \text{ infected by } j) \\
p_{i,I}(t + dt) &= p_{i,S}(t)[1 - \prod_{j \in \mathbf{H}_{-i}} (1 - \text{Prob. } i \text{ infected by } j)] + p_{i,I}(t)(1 - \text{Prob. recover}) \\
p_{i,R}(t + dt) &= p_{i,R}(t) + p_{i,I}(t)(\text{Prob. recover})
\end{aligned}
\tag{S27}
$$

The term Prob. i infected by j can be written as $1 - \exp(-p_{j,I}(t) FOI_{i \leftarrow j} dt)$, where $FOI_{i \leftarrow j}$ is the average force of infection i feels from j in the follow-the-leader model across the entire landscape. Prob. recover =

$1 - \exp(-\gamma dt)$, where γ is a per capita recovery rate.

We can approximate $\exp(-p_{j,I}(t)FOI_{i\leftarrow j}dt) \approx 1 - p_{j,I}(t)FOI_{i\leftarrow j}dt$ for small dt. The product $\prod_{j\in\mathbf{H}_{-i}}(1 - p_{j,I}(t)FOI_{i\leftarrow j}dt)$ can be expanded and dropping second order terms or higher we can write $\prod_{j\in\mathbf{H}_{-i}}(1 - p_{j,I}(t)FOI_{i\leftarrow j}dt) \approx 1 - \sum_{j\in\mathbf{H}_{-i}} p_{j,I}(t)FOI_{i\leftarrow j}dt$. Similarly, we can approximate Prob. recover $= \gamma dt$ for small dt. Ultimately, this allows us to write the approximate "mean-field" equation for the individual-level probabilities

$$\begin{aligned} p_{i,S}(t+dt) &= p_{i,S}(t)[1 - \sum_{j\in\mathbf{H}_{-i}} p_{j,I}(t)FOI_{i\leftarrow j}dt] \\ p_{i,I}(t+dt) &= p_{i,S}(t)\sum_{j\in\mathbf{H}_{-i}} p_{j,I}(t)FOI_{i\leftarrow j}dt + p_{i,I}(t)(1-\gamma dt) \\ p_{i,R}(t+dt) &= p_{i,R}(t) + p_{i,I}(t)\gamma dt \end{aligned} \tag{S28}$$

which we can further re-write as

$$\begin{aligned} \frac{dp_{i,S}(t)}{dt} &= -p_{i,S}(t)\sum_{j\in\mathbf{H}_{-i}} p_{j,I}(t)FOI_{i\leftarrow j} \\ \frac{dp_{i,I}(t)}{dt} &= p_{i,S}(t)\sum_{j\in\mathbf{H}_{-i}} p_{j,I}(t)FOI_{i\leftarrow j} - p_{i,I}(t)\gamma \\ \frac{p_{i,R}(t)}{dt} &= p_{i,I}(t)\gamma \end{aligned} \tag{S29}$$

This is a continuous-time, linear approximation of equation S27, and is useful for assessing how R_0 depends on correlated individual movement.

Before deriving R_0, we need to define $FOI_{i\leftarrow j}$. From the PMoveSTIR theory for direct transmission, random space use, and correlated movement (equation 8 in the main text), we know that average force of infection felt by i from j in location x is

$$h^*_{i\leftarrow j}(x) = \beta'\lambda\pi\eta\left[\frac{A_x}{A_{tot}}\frac{A_x}{A_{tot}} + \frac{A_x}{A_{tot}}(1 - \frac{A_x}{A_{tot}})\rho(x, s=0)\right]. \tag{S30}$$

For the follow-the-leader model, $A_x = (1/n_p)$ and $A_{tot} = N_h$ such that $\frac{A_x}{A_{tot}} = \frac{1}{N_h n_p}$. Letting $\beta'\lambda\pi\eta = \beta$ (strictly for notational simplicity), we have

$$h^*_{i\leftarrow j}(x) = \beta[\frac{1}{N_h n_p}\frac{1}{N_h n_p} + (\frac{1}{N_h n_p})(1 - \frac{1}{N_h n_p})\rho(x, s=0)]$$

Across the entire landscape consisting of $N_h n_p$ areas of transmission, $FOI_{i\leftarrow j} = h^*_{i\leftarrow j}(x)N_h n_p = \beta[\frac{1}{N_h n_p} + (1 - \frac{1}{N_h n_p})\rho(x, s=0)]$. We multiply by $N_h n_p$ because we are adding the FOI together across all possible locations of transmission on the landscape and they are the same across the entire landscape for the follow-

the-leader model.

With the functional form of $FOI_{i\leftarrow j}$, we can compute R_0 from equation S29 using a standard next generation matrix approach where R_0 is the spectral radius of $\mathbf{F}(-\mathbf{U})^{-1}$ [11]. For the follow-the-leader model and equation S29,

$$\mathbf{U} = \begin{bmatrix} -\gamma & 0 & \cdots & 0 \\ 0 & -\gamma & \cdots & 0 \\ \vdots & \vdots & \vdots & \vdots \\ 0 & 0 & \cdots & -\gamma \end{bmatrix} \tag{S31}$$

with dimension $H \times H$. $\mathbf{F}$ is also of dimension $H \times H$ and is given by

$$\mathbf{F} = FOI_{i\leftarrow j} \begin{bmatrix} 0 & 1 & 1 & \cdots & 1 \\ 1 & 0 & 1 & \cdots & 1 \\ \vdots & \vdots & \vdots & \vdots & \vdots \\ 1 & 1 & 1 & \cdots & 0 \end{bmatrix} \tag{S32}$$

We pull the $FOI_{i\leftarrow j}$ term out because all pairwise interactions in the follow-the-leader model have the same FOI. It is then easy to see that the spectral radius of $\mathbf{F}(-\mathbf{U})^{-1}$, and thus R_0, is

$$R_0 = \frac{(H-1)FOI_{i\leftarrow j}}{\gamma} = \frac{(H-1)\beta[\frac{1}{N_h n_p} + (1 - \frac{1}{N_h n_p})\rho(x, s=0)]}{\gamma}$$

By letting $n_p = 1$ (i.e., host social decisions are happening on the same scale as the area of transmission) and plugging in equation S26 for $\rho_{messy}(x, s = 0)$ we get the equation for R_0 for the follow-the-leader model with SIR dynamics as given in the main text.

7 Direct transmission and Ornstein-Uhlenbeck movement

Consider the Ornstein-Uhlenbeck process of movement with social correlation as described in the main text. Here, we illustrate how we can use this model to exactly calculate $CRS(x)$ for direct transmission.

Without loss of generality, let x be a grid cell with area $A_x = 100m^2$ bounded from -5 to 5 m along the x and y directions. Then marginal probability of host 1 using location x is

$$p_1(x) = \int_{x_1=-5}^{5} \int_{y_1=-5}^{5} \int_{x_2=-\infty}^{\infty} \int_{y_2=-\infty}^{\infty} MVN(\mathbf{x}|\boldsymbol{\mu}, \boldsymbol{\Lambda}) dx_1 dy_1 dx_2 dy_2$$

and host 2 using location x is

$$p_2(x) = \int_{x_1=-\infty}^{\infty} \int_{y_1=-\infty}^{\infty} \int_{x_2=-5}^{5} \int_{y_2=-5}^{5} MVN(\mathbf{x}|\boldsymbol{\mu}, \boldsymbol{\Lambda}) dx_1 dy_1 dx_2 dy_2$$

The joint probability of both host 1 and host 2 using location x at time lag $s = 0$ is

$$p_{12}(x) = \int_{x_1=-5}^{5} \int_{y_1=-5}^{5} \int_{x_2=-5}^{5} \int_{y_2=-5}^{5} MVN(\mathbf{x}|\boldsymbol{\mu}, \boldsymbol{\Lambda}) dx_1 dy_1 dx_2 dy_2$$

We can then define the pairwise correlation surface at time lag 0 $\rho(x, s = 0)$ as

$$\rho(x, s = 0) = \frac{p_{12}(x) - p_1(x)p_2(x)}{\sigma_1(x)\sigma_2(x)}$$

for any location x, where $\sigma_1(x) = \sqrt{p_1(x)(1 - p_1(x))}$ and $\sigma_2(x) = \sqrt{p_2(x)(1 - p_2(x))}$

We illustrate these calculations for two hosts moving according to an OU process with $\sigma = 150m$, social attraction $\xi = 0.9$, and home range centers $\boldsymbol{\mu} = [0\ 0\ 0\ 0]'$ (ignoring c for the moment as this rate does not matter for the long-term stationary probability). The value of σ is chosen such that the 95%-100% home range sizes are approximately 0.3 km^2 - 1 km^2, similar to what we might expect for female white-tailed deer in western Tennessee, USA. The marginal UDs for host 1 and host 2 are obviously identical and are shown in Fig. S1A computed across a landscape-level grid with 10m by 10m grid cells ($A_x = 100m^2$). Fig. S1B shows the pairwise correlation surface $\rho(x, s = 0)$ across the gridded landscape. While there are some noticeable spatial patterns apparent in the correlation surface (this is because the variance in space use is decreasing more quickly then the covariance in space use as we move away from the home range center), the correlation surface is similar across this portion of the landscape considered here – $\rho(x, s = 0) \approx 0.0028$ for all 10m by 10m locations x on the landscape (ranging from between 0.0018 - 0.0032 across this portion of landscape). Finally, from these two surfaces we can compute the $CSR(x)$ for direct contact: $\sigma_1(x)\sigma_2(x)\rho(x, s = 0)/p_1(x)p_2(x)$ (Fig. S1C). We see that across the entire landscape non-independent movement is contributing orders of magnitude more to FOI then spatial overlap and that the greatest relative effect of non-independent movement to FOI comes away from the home range center (Fig. S1C). For areas with higher joint, marginal use – such as the home range center – it will matter less for FOI whether additional social factors are also pulling individuals into these areas. Thus, FOI is driven more by spatial overlap in areas with higher probability of use.

8 Indirect transmission and Ornstein-Uhlenbeck movement

The OU process we use in the main text can be written as [12]

$$\mathbf{x}(t+\Delta t) \sim MVN(\boldsymbol{\mu} + e^{-\mathbf{B}\Delta t}(\mathbf{x}(t) - \boldsymbol{\mu}), \boldsymbol{\Lambda} - e^{-\mathbf{B}\Delta t}\boldsymbol{\Lambda}e^{-\mathbf{B}'\Delta t}) \tag{S33}$$

where MVN specifies a multivariate normal distribution. We can recognize equation S33 as the conditional distribution of a multivariate normal distribution with the following form

$$[\mathbf{x}_1|\mathbf{x}_2 = \mathbf{a}] = MVN(\boldsymbol{\mu}_1 + \boldsymbol{\Sigma}_{12}\boldsymbol{\Sigma}_{22}^{-1}(\mathbf{a} - \boldsymbol{\mu}_2), \boldsymbol{\Sigma}_{11} - \boldsymbol{\Sigma}_{12}\boldsymbol{\Sigma}_{22}^{-1}\boldsymbol{\Sigma}_{21})$$

where the joint MVN for $\begin{bmatrix}\mathbf{x}_1 & \mathbf{x}_2\end{bmatrix}'$ has a mean $\begin{bmatrix}\boldsymbol{\mu}_1 & \boldsymbol{\mu}_2\end{bmatrix}'$ and a variance-covariance matrix of

$$\begin{bmatrix}\boldsymbol{\Sigma}_{11} & \boldsymbol{\Sigma}_{12} \\ \boldsymbol{\Sigma}_{21} & \boldsymbol{\Sigma}_{22}\end{bmatrix}$$

We can then write the joint distribution for $\mathbf{x}_1 = \mathbf{x}(t+\Delta t)$ and $\mathbf{x}_2 = \mathbf{x}(t)$ as

$$\begin{bmatrix}\mathbf{x}(t+\Delta t) \\ \mathbf{x}(t)\end{bmatrix} \sim MVN(\begin{bmatrix}\boldsymbol{\mu}_1 \\ \boldsymbol{\mu}_2\end{bmatrix}, \begin{bmatrix}\Lambda & e^{-\mathbf{B}\Delta t}\Lambda \\ e^{-\mathbf{B}\Delta t}\Lambda & \Lambda\end{bmatrix}) \tag{S34}$$

Here, $\boldsymbol{\mu}_1$ and $\boldsymbol{\mu}_2$ are identical vectors and are the home range centers for the hosts. The matrix $\mathbf{B} = c\mathbf{I}$ is the diagonal matrix of drift coefficients given in the main text and $\boldsymbol{\Lambda}$ is the variance-covariance matrix.

From the main text, recall that the vector $\mathbf{x}(t)$ is a 4×1 vector that contains the locations of both host 1 and host 2. Thus, $\boldsymbol{\Lambda}$ is a 4×4 matrix that contains a standard deviation parameter σ and a social attraction parameter ξ that induced correlation between hosts. For simplicity, we assume that parameters do not differ among hosts.

Armed with equation S34, we can now exactly calculate the probability of host 1 at time $t + \Delta t = t'$ and host 2 at time t both being in location x, $p_{1 \text{ at } t', 2 \text{ at } t}(x)$. Without loss of generality, say that location x is the area of transmission with $A_x = 100m^2$ bounded by -5 and 5 m in both the x and y direction, then

$$p_{1 \text{ at } t', 2 \text{ at } t}(x) = \int_{-5}^{5}\int_{-5}^{5}\int_{-\infty}^{\infty}\int_{-\infty}^{\infty}\int_{-\infty}^{\infty}\int_{-\infty}^{\infty}\int_{-5}^{5}\int_{-5}^{5} MVN(\begin{bmatrix}\mathbf{x}(t') \\ \mathbf{x}(t)\end{bmatrix} | \begin{bmatrix}\mu_1 \\ \mu_2\end{bmatrix}, \begin{bmatrix}\Lambda & e^{-\mathbf{B}\Delta t}\Lambda \\ e^{-\mathbf{B}\Delta t}\Lambda & \Lambda\end{bmatrix}) dx_{1,t'}dy_{1,t'}dx_{2,t'}dy_{2,t'}dx_{1,t}dy_{1,t}dx_{2,t}dy_{2,t}$$

This equation looks intimidating, but it is just a specific marginal over a multivariate normal distribution and is easy and fast to compute (we used the `multivariate_normal` in Python's scipy). We can then calculate

$\rho(x, s = \Delta t)$ as

$$\rho(x, s = \Delta t) = \frac{p_{1 \text{ at } t', 2 \text{ at } t}(x) - p_1(x)p_2(x)}{\sigma_1(x)\sigma_2(x)}$$

where we recognize that given stationarity, $p_i(x)$ and $\sigma_i(x)$ do not depend on t or t'.

References

[1] Wilber MQ, Yang A, Boughton R, Manlove KR, Miller RS, Pepin KM, et al. A model for leveraging animal movement to understand spatio-temporal disease dynamics. Ecology Letters. 2022;25(5):1290–1304.

[2] Dwyer G, Elkinton JS, Buonaccorsi JP. Host Heterogeneity in Susceptibility and Disease Dynamics: Tests of a Mathematical Model. The American Naturalist. 1997;150(6):685–707.

[3] Fenton A, Streicker DG, Petchey OL, Pedersen AB. Are all hosts created equal? Partitioning host species contributions to parasite persistence in multihost communities. The American Naturalist. 2015;186:610–622.

[4] Arino J, van den Driessche P. Time delays in epidemic models: modeling and numerical considerations. In: Delay Differential Equations and Applications. Springer; 2006. p. 539–578.

[5] Gurarie E, Ovaskainen O. Towards a general formalization of encounter rates in ecology. Theoretical Ecology. 2013;6(2):189–202.

[6] Martinez-Garcia R, Fleming CH, Seppelt R, Fagan WF, Calabrese JM. How range residency and long-range perception change encounter rates. Journal of Theoretical Biology. 2020;498:110267.

[7] Grimmett G, Stirzaker D. Probability and random processes. Oxford ;: Oxford University Press; 2001.

[8] Hooten MB, Johnson DS, McClintock BT, Morales JM. Animal Movement: Statistical Models for Telemetry Data. New York, USA: CRC Press; 2017.

[9] Ahn HJ, Hassibi B. Global dynamics of epidemic spread over complex networks. Proceedings of the IEEE Conference on Decision and Control. 2013;(1):4579–4585.

[10] Ruhi NA, Hassibi B. SIRS epidemics on complex networks: Concurrence of exact Markov chain and approximated models. Proceedings of the IEEE Conference on Decision and Control. 2015;54rd IEEE(Cdc):2919–2926.

[11] Diekmann O, Heesterbeek JAP, Britton T. Mathematical Tools for Understanding Infectious Disease Dynamics. Princeton: Princeton University Press; 2013.

[12] Blackwell PG. Random diffusion models for animal movement. Ecological Modelling. 1997;100(1-3):87–102.

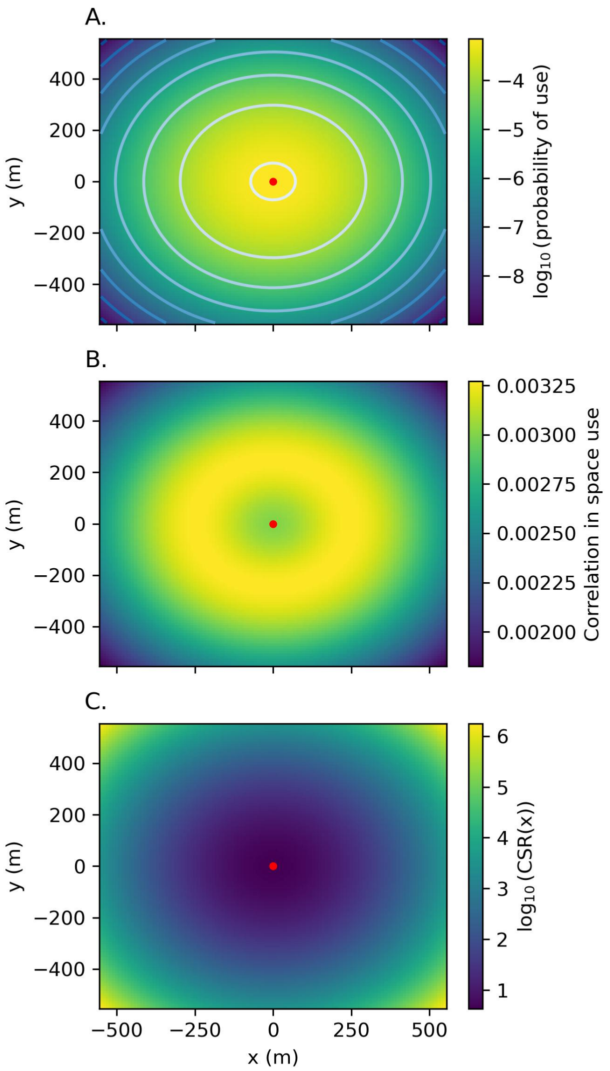

Figure S1: **A.** The stationary utilization distribution (UD) of a host following an Ornstein-Uhlenbeck (OU) process with $\sigma = 150m$, social attraction $\xi = 0.9$, and an attracting home range center at $x = 0$ and $y = 0$. Probabilities of use are computed from the UD over 10m $\times$ 10m grid cells. Contours illustrate the elliptical nature of the OU UD, consistent with a multivariate normal distribution. **B.** The correlation surface for two individuals with social attraction $\xi = 0.9$ moving according to the OU process. **C.** The relative contribution of non-independent movement to FOI compared to spatial overlap (the ratio between these two quantities). On all plots, the red dot indicates the home range center $\boldsymbol{\mu}$.
